# Actinobacteria from Antarctica as a source for anticancer discovery

**DOI:** 10.1038/s41598-020-69786-2

**Published:** 2020-08-17

**Authors:** Leonardo Jose Silva, Eduardo José Crevelin, Danilo Tosta Souza, Gileno Vieira Lacerda-Júnior, Valeria Maia de Oliveira, Ana Lucia Tasca Gois Ruiz, Luiz Henrique Rosa, Luiz Alberto Beraldo Moraes, Itamar Soares Melo

**Affiliations:** 1 College of Agriculture “Luiz de Queiroz”, University of São Paulo (USP), Piracicaba, SP Brazil; 2 Laboratory of Mass Spectrometry Applied To Natural Products Chemistry, Department of Chemistry, Faculty of Philosophy, Sciences and Letters of Ribeirão Preto (FFCLRP), University of São Paulo (USP), Ribeirão Preto, SP Brazil; 3 Laboratory of Environmental Microbiology, Brazilian Agricultural Research Corporation (EMBRAPA) – Embrapa Environment, Jaguariúna, SP Brazil; 4 Microbial Resourses Division, Research Center for Chemistry, Biology and Agriculture (CPQBA), University of Campinas (UNICAMP), Campinas, SP Brazil; 5 Faculty of Pharmaceutical Sciences, University of Campinas (UNICAMP), Campinas, SP Brazil; 6 Department of Microbiology, Biological Sciences Institute – Federal University of Minas Gerais (UFMG), Belo Horizonte, MG Brazil

**Keywords:** Biotechnology, Sequencing, High-throughput screening

## Abstract

Although many advances have been achieved to treat aggressive tumours, cancer remains a leading cause of death and a public health problem worldwide. Among the main approaches for the discovery of new bioactive agents, the prospect of microbial secondary metabolites represents an effective source for the development of drug leads. In this study, we investigated the actinobacterial diversity associated with an endemic Antarctic species, *Deschampsia antarctica*, by integrated culture-dependent and culture-independent methods and acknowledged this niche as a reservoir of bioactive strains for the production of antitumour compounds. The 16S rRNA-based analysis showed the predominance of the Actinomycetales order, a well-known group of bioactive metabolite producers belonging to the Actinobacteria phylum. Cultivation techniques were applied, and 72 psychrotolerant Actinobacteria strains belonging to the genera *Actinoplanes*, *Arthrobacter*, *Kribbella*, *Mycobacterium*, *Nocardia*, *Pilimelia*, *Pseudarthrobacter*, *Rhodococcus*, *Streptacidiphilus*, *Streptomyces* and *Tsukamurella* were identified. The secondary metabolites were screened, and 17 isolates were identified as promising antitumour compound producers. However, the bio-guided assay showed a pronounced antiproliferative activity for the crude extracts of *Streptomyces* sp. CMAA 1527 and *Streptomyces* sp. CMAA 1653. The TGI and LC_50_ values revealed the potential of these natural products to control the proliferation of breast (MCF-7), glioblastoma (U251), lung/non-small (NCI-H460) and kidney (786-0) human cancer cell lines. Cinerubin B and actinomycin V were the predominant compounds identified in *Streptomyces* sp. CMAA 1527 and *Streptomyces* sp. CMAA 1653, respectively. Our results suggest that the rhizosphere of *D. antarctica* represents a prominent reservoir of bioactive actinobacteria strains and reveals it as an important environment for potential antitumour agents.

## Introduction

In general, microbial natural products have higher selectivity and bioactivity indices when compared to combinatorial chemistry libraries^1,2^. The search for bioactive molecules from microorganisms has received growing attention in recent decades^3–5^. Part of this is due to the specific action of microbial metabolites as substrates for many transport systems, which allows the release of compounds into intracellular sites^6,7^. However, despite the extensive search for microbial metabolites to address a great deal of clinical threat, the discovery rate of new and effective drug compounds has been declining every year, which is due, in part, to the use of traditional techniques of chemical isolation and the investigation of microorganisms in extensively studied environments^3,8,9^.

Thus, bioprospecting studies have advanced into auspicious ecological niches, which tend to favour the prevalence of exotic metabolisms and endemic species^10,11^. In this context, the Antarctic continent has been considered one of the most promising bioprospecting ecosystems and a valuable source to isolate new and diverse microorganisms due to its environmental peculiarities, such as extremely low temperatures and precipitation, high levels of UV radiation, ocean flooding, high salinity rates, and large unexplored areas^12–16^.

Although many organisms are adapted to the harshest environmental conditions of the Antarctic, the species *Deschampsia antarctica* Desv. (Poaceae) and *Colobanthus quitensis* (Kunth) Bartl. (Caryophyllaceae) have received more attention because they represent unique vascular plants in the whole Antarctic continent and play an important ecological role as a shelter for a plethora of microbes with wide metabolic capacities^17,18^. However, the reduced dispersion of *D. antarctica* Desv. in few Antarctic sites has attracted special interest in ecological and bioprospecting investigations^17,19,20^.

In this way, several studies have been carried out to characterize the microbiome associated with Antarctic hairgrass (*D. antarctica*), as well as to understand the adaptive mechanisms of this species to survive the harsh environmental conditions of Antarctica^21–23^. Even so, studies exploring the potential of this microbiome to access novel cultivable strains and bioactive compounds are still scarce.

**Figure 1:**
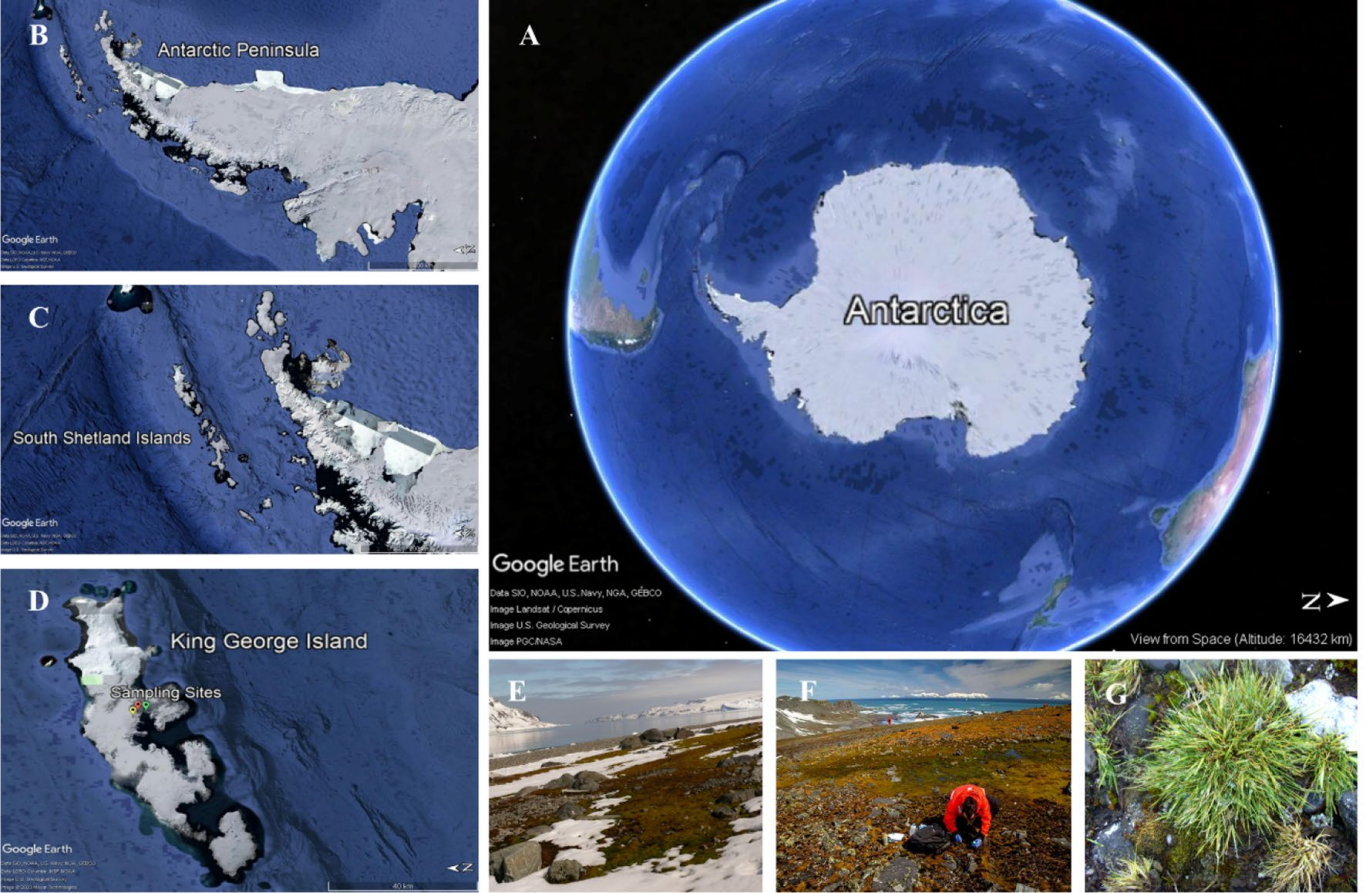
Satellite images (Google Earth Pro, 2019) and study sites. (**A**) Antarctic Continent; (**B**) Antarctic Peninsula; (**C**) South Shetland Islands; D-E–F) King George Island [sampling sites: Torre Meteoro Direito—TMD, Torre Meteoro Esquerdo—TME and Morro da Cruz—MDC (green, red and yellow location icon, respectively)]; (**G**) *Deschampsia antarctica* Desv.

Recently, Sivalingam and colleagues (2019)^24^ reported the prominence of Actinobacteria, especially species of *Streptomyces,* derived from extreme sources as an extraordinary reservoir of novel biosynthetic gene clusters with potential for developing anticancer drugs. Indeed, actinomycetes have been recognized as a prolific source of natural products with a myriad of bioactivities, including phytotoxic, antimicrobial, insecticidal and mainly antiproliferative and antitumour activities^25–28^. Compound classes belonging to the peptides, polyketides, macrolides, quinolones and others represent these bioactivities^28–32^. Accordingly, the isolation and identification of actinomycetes have become a fruitful area of research in recent years that has subsequently led to the identification of novel Actinomycete species that should be exploited to unveil possible biosynthetic pathways and discover new bioactive natural products^12,33–35^. Thus, the rhizosphere bacterial composition of Antarctic hairgrass (Fig. 1) was investigated by the 16S rRNA gene sequencing and the cultivable associated actinobacteria were evaluated as potential sources of antitumour compounds.

## Results

### Bacterial community associated with *Deschampsia antarctica* rhizosphere

**Figure 2:**
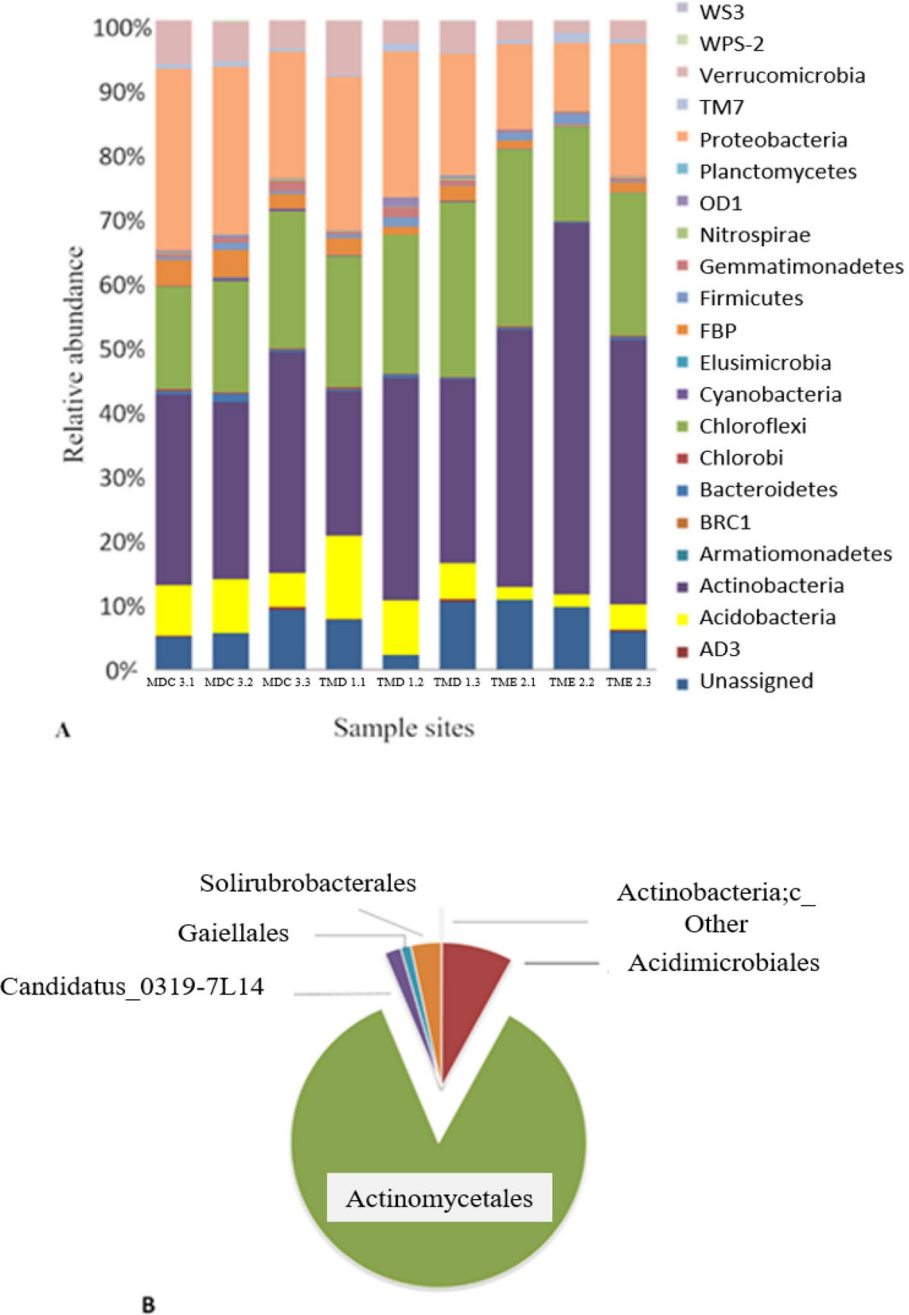
Taxonomic composition of *Deschampsia antarctica* rhizosphere collected across three sites revealed by 16S rRNA marker sequencing. (**A**) Bacteria phylum; (**B**) Order-level taxonomic affiliation of OTUs filtered from Actinobacteria phylum (Caporaso et al., 2011; Caporaso et al., 2010)^36,37^.

In this study, a total of 249.176 high-quality 16S rRNA reads were recovered after the QC filter step (Table S1—Supplementary Material). Sequences were assigned to 229.631 OTUs, and the rarefaction curves reached the plateau phases, confirming the adequate sequencing depth of the samples. The bacterial community structure revealed by the higher-ranking taxa classification was congruent among the different rhizosphere samples (Fig. 2).

The average taxonomic signatures of all samples from sites Morro da Cruz, Torre Meteoro Direito e Torre Meteoro Esquerdo (MDC, TMD and TME, respectively) showed that Actinobacteria (34%) was the most abundant phylum, followed by Chloroflexi (21%) and Proteobacteria (20%) (Fig. 2a). Members of the phyla Acidobacteria and Verrucomicrobia were detected at lower frequencies (< 5%). The ‘unclassified’ sequences contributed to 7% of the dataset, showing that the understanding of the plant-microbiome interaction is still limited and that the rhizosphere of *D. antarctica* may harbour unknown taxonomic groups to be further explored. A more detailed taxonomic analysis showed that the phylum Actinobacteria was mainly represented by members of the order Actinomycetales (86%), followed by Acidimicrobiales (8%) and Solirubrobacteriales (3%) (Fig. 2b).

### Functional prediction of the bacterial communities

**Figure 3:**
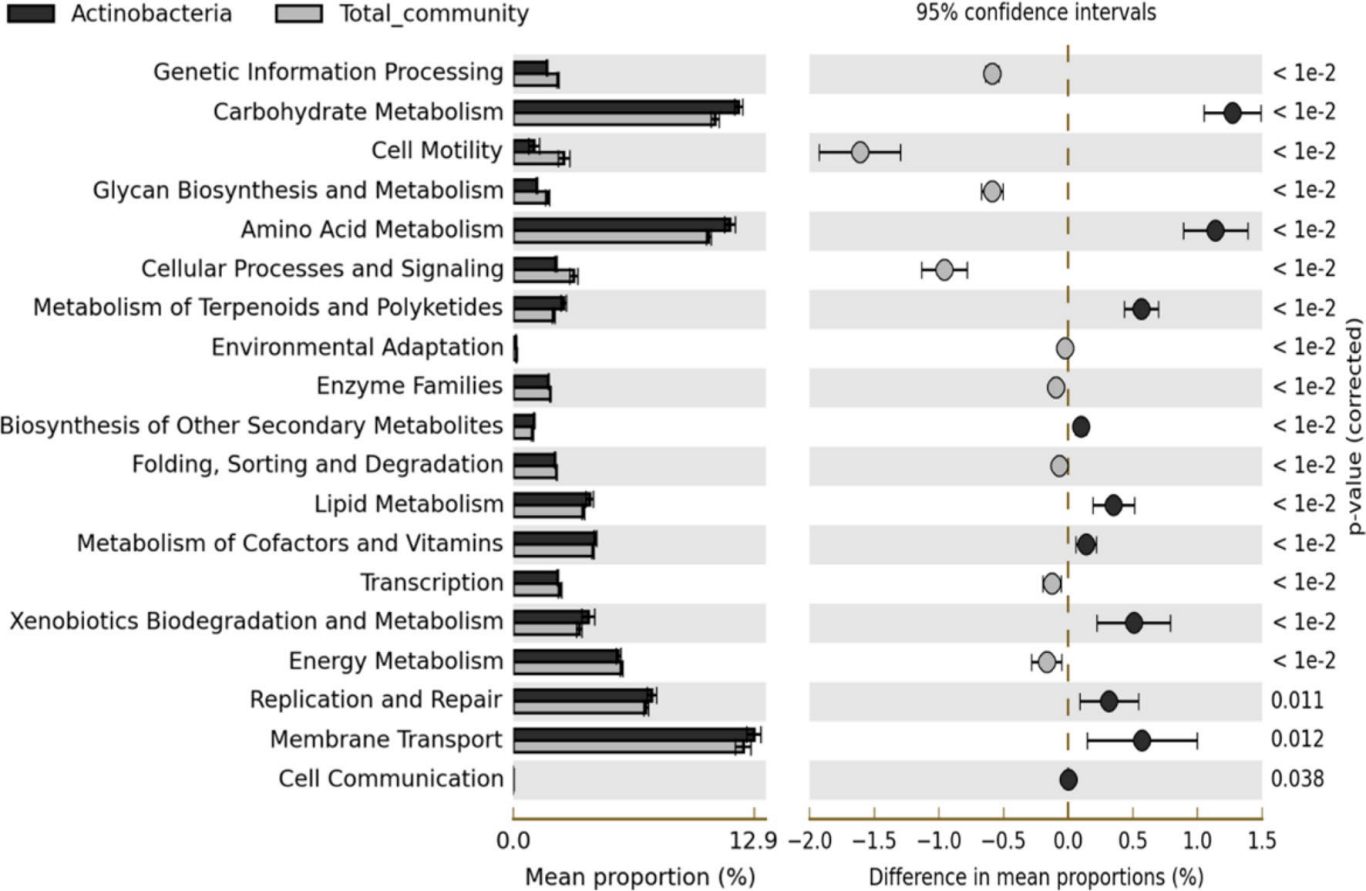
Extended error bar plot showing the predicted KO-level 2 categories significantly different between total community and Actinobacteria members from *Deschampsia antarctica* Desv. rhizosphere (n = 9). Only P < 0.05 is displayed. (Kanehisa; Goto, 2000; Kanehisa et al., 2019; Kanehisa, 2019; Langille et al., 2013)^38–41^.

The predictive functional profile of the entire rhizosphere-associated bacterial community resulted in more than 6,500 protein-coding genes, which could be assigned to 329 KEGG Orthology functional categories (KOs). To determine a specific functional contribution, Actinobacteria-affiliated 16S rRNA reads were extracted and compared with the predictive functional traits of the total bacterial community. Principal component analysis (PCA) showed a clear separation between the predicted functional profiles of the total bacterial community and Actinobacteria groups (Figure S1—Supplementary Material). Nineteen KOs were significantly over-represented (p-value < 0.05) between the two datasets. The pathways carbohydrate metabolism, amino acid metabolism, metabolism of terpenoids and polyketides, xenobiotic biodegradation and metabolism, DNA replication and repair, membrane transporter, and biosynthesis of other secondary metabolites were significantly enriched (p < 0.05) in the Actinobacteria dataset when compared to the total bacterial community (Fig. 3).

### Isolating, culturing and identifying actinobacteria

**Figure 4:**
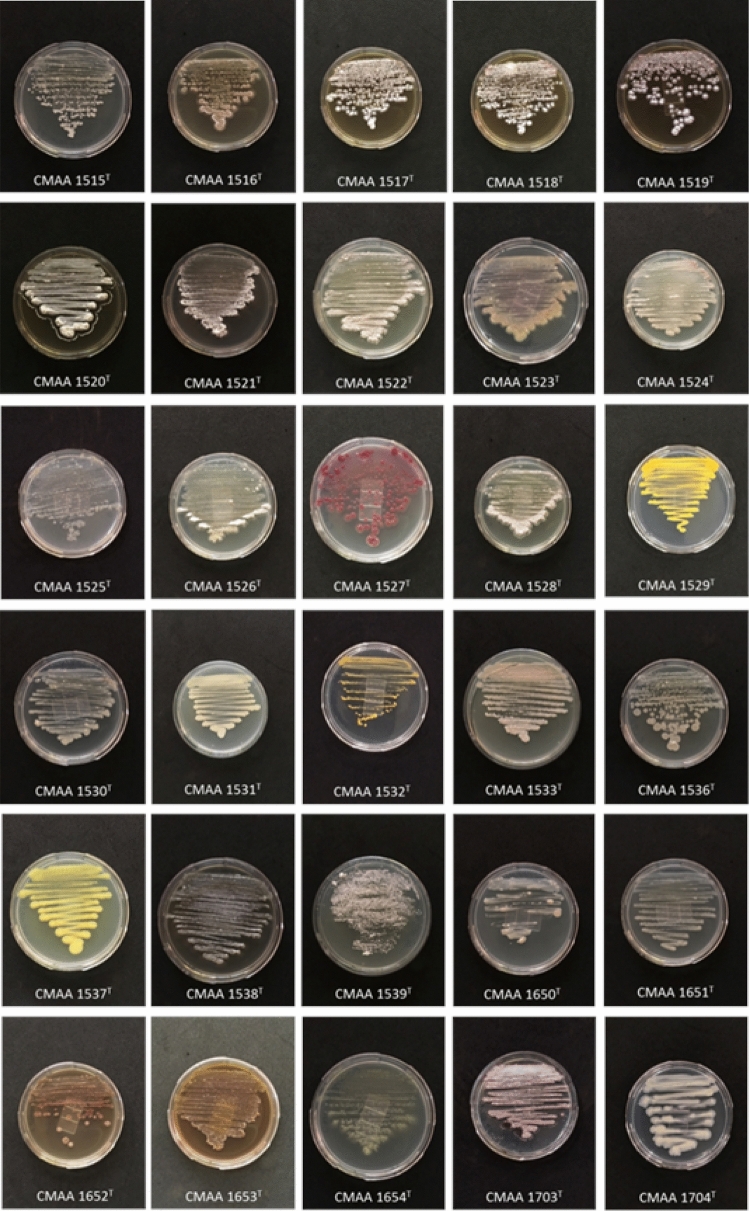
Morphological diversity of Actinobacteria strains isolated from the rhizosphere of *Deschampsia antarctica* Desv.

A total of 72 actinomycetes were isolated with the media and procedures employed in this study. The isolates belonged to the genera *Actinoplanes*, *Arthrobacter*, *Kribbella*, *Mycobacterium*, *Nocardia*, *Pilimelia*, *Pseudarthrobacter*, *Rhodococcus*, *Streptacidiphilus*, *Streptomyces* and *Tsukamurella* from 6 families in the phylum Actinobacteria. Genomic analysis based on 16S rRNA sequences was used to select 30 isolates as representative strains of the accessed actinomycetes diversity. The nearest type strains, the percentage of identity and the GenBank access number are presented in Table S2—Supplementary Material. The strains are deposited in the Collection of Microorganisms of Agricultural and Environment Importance (CMAA)—Embrapa, Brazil (Fig. 4).

### Screening for bioactive strains

**Figure 5:**
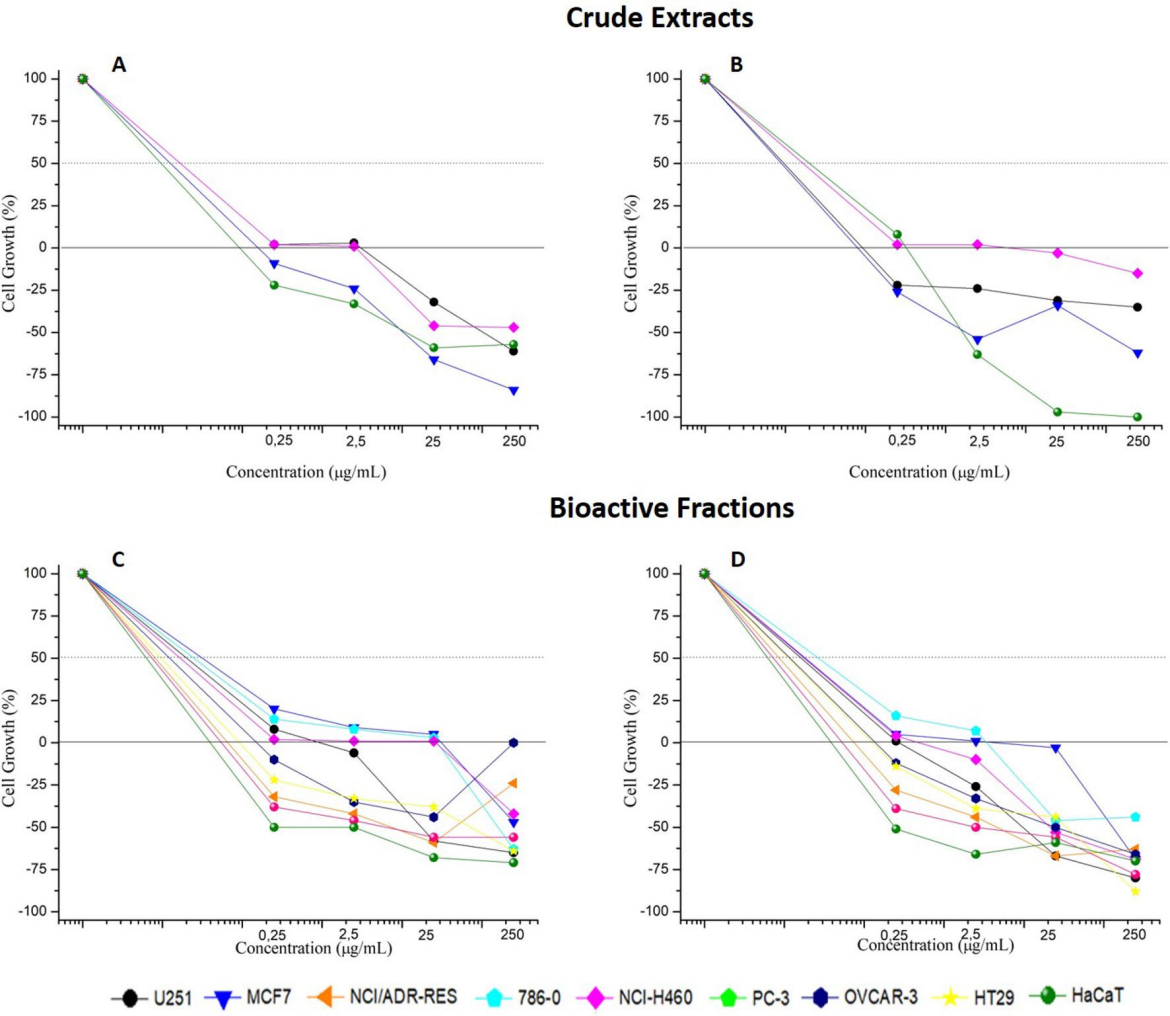
Antiproliferative activity in tumor cell lines. (**A**) *Streptomyces* sp. CMAA 1527 crude extract; (**B**) *Streptomyces* sp. CMAA 1653 crude extract; (**C**) Fraction FR-3 (produced by *Streptomyces* sp. CMAA 1527) and (**D**) Fraction FR-7 (produced by *Streptomyces* sp. 1653).

First, all thirty isolated strains were evaluated against *Pythium aphanidermatum* CMAA 243^T^. Seventeen strains showed growth inhibition effects, as determined by the antagonism test (Figure S2 and Table S3—Supplementary Material), and these strains were selected for the antiproliferative assay against human tumour cell lines; glioma (U251), breast 174 (MCF-7) and lung/non-small cells (NCI-H460). Two strains, CMAA 1527 and CMAA 1653, exhibited potent activity, considering that Total Growth Inhibition (TGI) values lower than 6.25 µg/mL. Crude extracts of both CMAA 1527 and CMAA 1653 completely inhibited the growth of breast (MCF-7, TGI < 0.25 µg/mL, both extracts), glioblastoma (U251, TGI = 3.05 and < 0.25 µg/mL, respectively) and non-small lung (NCI-H460, TGI = 0.57 and 5.8 µg/mL, respectively) tumour cells (Fig. 5).

### Phylogenetic analysis

**Figure 6:**
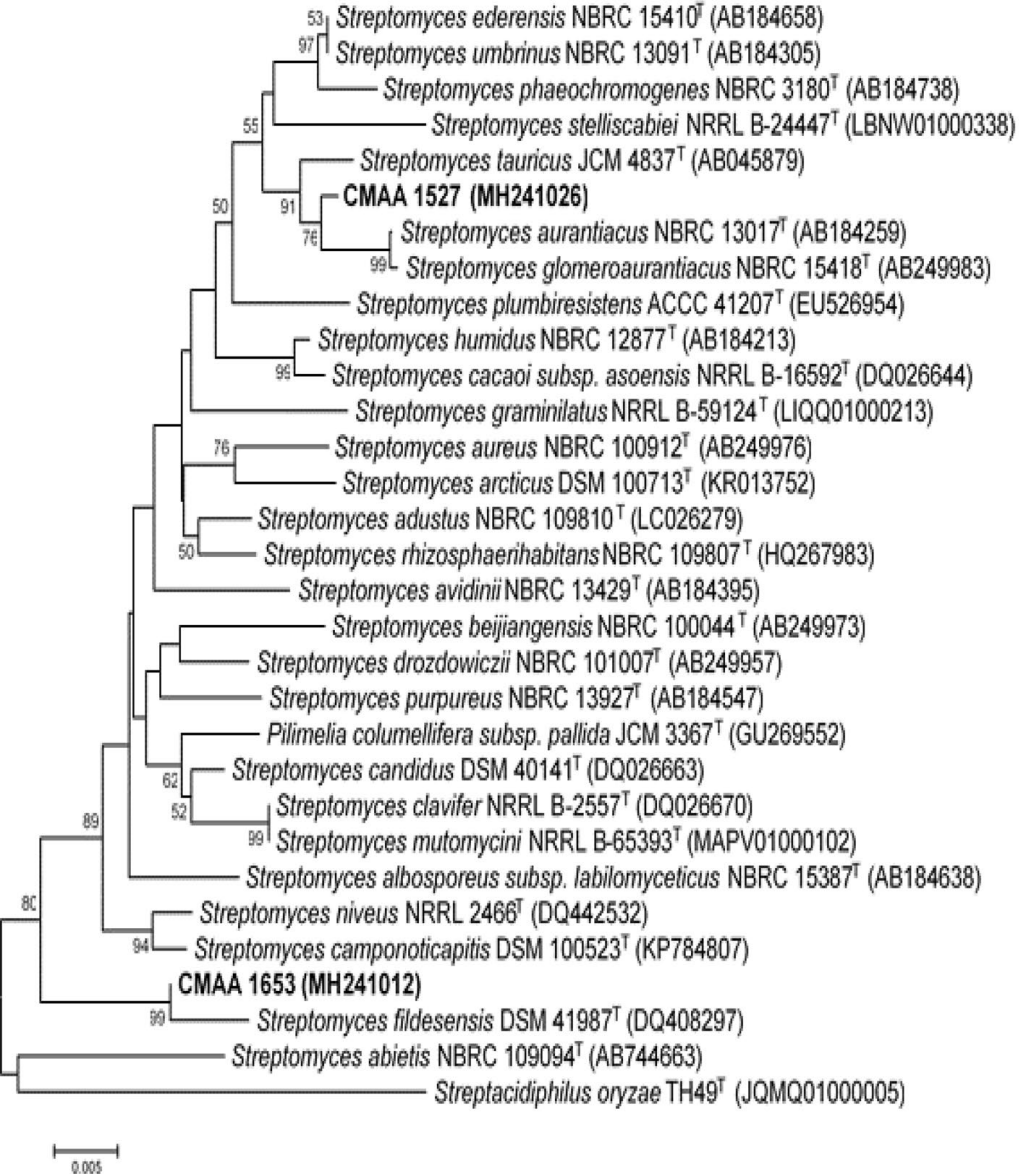
Phylogenetic tree based on the 16S rRNA gene sequences, obtained by Neighbor–Joining method analysis for the bioactive strains CMAA 1527 and CMAA 1653 and their closely related type strains—MEGA 7.0^42^. Numbers at nodes indicate the level of bootstrap support based on 1,000 replications (only values > 50% are shown). *Streptacidiphilus oryzae* was used as outgroup for this study.

According to the phylogenetic reconstruction based on the 16S rRNA gene similarity the bioactive actinobacterial strains were affiliated with the genus *Streptomyces*. The CMAA 1527 strain was closely related to *Streptomyces aurantiacus* NBRC 13017^T^, while CMAA 1653 was related to *Streptomyces fildesensis* DSM 41987^T^ (Fig. 6), although they form a distinct phyletic branch towards the phylogenetic tree.

### Bioassay-guided fractionation and structural identification of compounds

**Figure 7:**
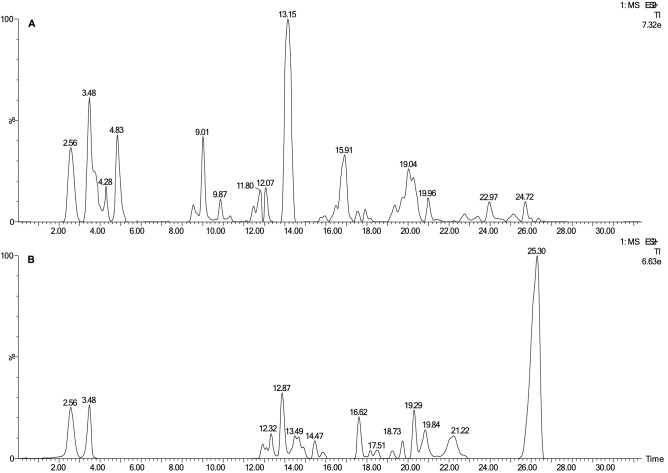
Chromatogram TIC obtained by LC–MS of crude extracts: (**A**) *Streptomyces* sp. CMAA 1527 and (**B**) *Streptomyces* sp. CMAA 1653.

The crude extracts obtained from *Streptomyces* sp. CMAA 1527 and *Streptomyces* sp. CMAA 1653 showed high antiproliferative activity. Both extracts were analysed by LC–MS and presented different chemical profiles (Fig. 7).

The major peak in *Streptomyces* sp. CMAA 1527 crude extract was analysed by LC–MS at the retention time of 13.15 min and had mass spectra with *m/z* 826 (Fig. 7a). The extract was fractionated by semi-preparative HPLC, resulting in 4 grouped fractions (FR-1, FR-2, FR-3 and FR-4) that were subjected to a biological assay. Figure 5c and Table 1 shows the antiproliferative potential of fraction FR-3, the most bioactive fraction according to human cancer cell line panel. FR-3 was submitted to structural identification experiments, although other fractions showed lower biological activities (data not shown).

**Table 1:** In vitro antiproliferative activity of crude extracts and bioactive fractions against human tumor and non-tumor cell lines.

	Cell lines								
Crude extracts and bioactive fractions	1	2	3	4	5	6	7	8	9
	**TGI (μg/ml)**^**b**^								
**Doxorubicin**^**a**^	0.56 ± 0.42 (P)	0.42 ± 0.11 (P)	2.3 ± 0.9 (P)	4.2 ± 4.3 (P)	* *(P)	1.3 ± 0.4 (P)	3.6 ± 1.8 (P)	2.4 ± 0.8 (P)	0.16 ± 0.04 (P)
CMAA 1527	3.05 ± 0.09 (P)	* * (P)	ND	ND	0.57 ± 0.67 (P)	ND	ND	ND	* * (P)
CMAA 1653	* * (P)	* * (P)	ND	ND	5.8 ± 3.3 (P)	ND	ND	ND	0.31 ± 0.03 (P)
Fraction FR-3	0.65 ± 0.43 (P)	5.7 ± 5.7 (P)	* * (P)	3.2 ± 4.2 (P)	1.5 ± 2.6 (P)	* * (P)	* * (P)	* * (P)	* * (P)
Fraction FR-7	0.31 ± 0.11 (P)	1.4 ± 2.3 (P)	* * (P)	1.5 ± 1.3 (P)	0.51 ± 0.27 (P)	* * (P)	* * (P)	* * (P)	* *(P)
	**LC**_**50**_** (μg/ml)**^**c**^								
**Doxorubicin**^**a**^	> 25	> 25	> 25	> 25	> 25	> 25	> 25	25	1.7 ± 1.6
Fraction FR-3	38.8 ± 31.6	*	*	*	*	*	54.1 ± 33.4	11.2 ± 6.7	0.51 ± 0.54
Fraction FR-7	12.2 ± 4.9	162.6 ± 23.7	6.7 ± 3.5^#^	*	38.1 ± 20.2^#^	28.8 ± 8.2^#^	13.7 ± 9.6	2.6 ± 1.5	* *

(a) Doxorubicin: chemotherapeutic drug; (b) TGI: Total Growth Inhibition (sample concentration required for total cell growth inhibition) and (c) LC_50_: Lethal Concentration 50 (sample concentration required to elicit 50% of cell death), expressed in µg/ml followed by standard error, calculated by sigmoidal regression using Origin 8.0 software. Results classified according to NCI’s criteria based on TGI values (I, inactive sample: TGI > 50 μg/ml; W, weak activity: 15 μg/ml < TGI < 50 μg/ml; M, moderate activity: 6.25 μg/ml < TGI < 15 μg/ml; P, potent activity: TGI < 6.25 μg/ml)^43^. LC_50_ results were compared by Test t-student [#: statistically significant difference (p ≤ 0.05) between fractions FR-3 and FR-7 in the same cell line]. Human tumor cell lines: 1 = U251 (glioblastoma); 2 = MCF-7 (breast, adenocarcinoma); 3 = NCI/ADR-RES (ovary, multi-drug resistant adenocarcinoma), 4 = 786–0 (kidney, adenocarcinoma), 5 = NCI-H460 (lung, large cell carcinoma); 6 = OVCAR-3 (ovary, adenocarcinoma), 7 = HT-29 (colon, adenocarcinoma), 8 = K-562 (chronic myeloid leukemia); Human non tumor cell line: 9 = HaCaT (immortalized keratinocyte); (*) effective concentration (TGI or GI_50_) > 250 μg/mL; (* *) effective concentration (TGI or GI_50_) < 0.25 μg/mL; (ND) not determined.

**Figure 8:**
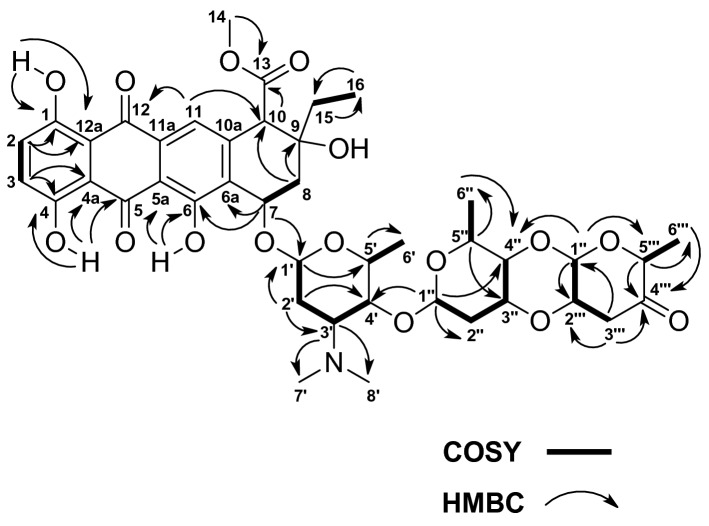
The main HMBC and COSY correlations observed for cinerubin B.

The chemical structure of FR-3 was confirmed by the analysis of the HRESIMS spectrum, and the molecular formula was determined to be C_42_H_51_NO_16_ as [M + H]^+^ was observed at *m/z* 826.3298 u, calculated for C_42_H_52_NO_16_^+^ (Figure S3—Supplementary Material). Cinerubin B was identified based on spectral data such as HRESIMS, ^1^H NMR, COSY, gHMBC, and gHMQC. The compound was isolated as a red amorphous powder that was soluble in methanol (MeOH) and trichloromethane (CHCl_3_) with UV absorption maxima at 490 nm. The ^1^H NMR spectrum of cinerubin B (Figure S4—Supplementary Material) showed signs of characteristic chemical shifts of anthracycline class; the signals of phenolic hydrogen were at δ 12.99 (s, 1H), δ 12.83 (s, 1H), and δ 12.28 (s, 1H). There were also signs of aromatic hydrogen at δ 7.75 (s, 1H) and δ 7.33 (d, *J* = 4.41 Hz, 2 H), as well as a broad doublet for a carbinolic hydrogen at δ 5.27 (dl, *J* = 2.50 Hz, 1 H). Other observed signals that are characteristic of this compound refer to three methoxyl group hydrogens at δ 3.71 (s, 3H), the hydrogen signals from an ethyl group at δ 1.76 (q, *J* = 7.0 Hz, 2H) and δ 1.09 (t, *J* = 7.0 Hz, 3H), and the methyl hydrogen signals belonging to an *N*-dimethyl group at δ 2.16 (s, 6H). In addition, the presence of three anomeric hydrogens at δ 5.49 (sl, 1H), δ 5.12 (dl, *J* = 2.9 Hz, 1H), and δ 5.21 (dl, *J* = 2.9 Hz, 1H) indicated the presence of a trisaccharide moiety in the chemical structure of the compound. The 2D correlations from the HMBC and COSY spectra (Table S4—Supplementary Material) were used to assign all signals present in the cinerubin B structure (Fig. 8 and Figure S5—Supplementary Material).

In order to identify other anthracyclines the LC–MS/MS analysis using neutral loss methodology was performed. The chemical structure of the predominant compound produced by *Streptomyces* sp. CMAA 1527 (cinerubin B; *m/z* 826.3298 u) showed a neutral loss of 240 u, which refers to a charge-remote hydrogen rearrangement with consequent loss of disaccharide cinerulosyl-2-deoxyfucosyl to form the product ion *m/z* 586 as a base peak, which it was attributed to pyrromycin. This methodology allowed us to show the presence of other bioactive compounds in the crude extract (Figure S6—Supplementary Material) but with lower concentrations than cinerubin B since it was not possible to perform the isolation process. These compounds were identified as 1 or 11-hydroxysulfurmycin B (*m/z* 854), auramycin B (*m/z* 812) or *N*-desmethylcinerubine B (*m/z* 812) and 1-deoxycinerubin B (*m/z* 810) which have been attributed to the lower bioactivities detected at the others fractions (Figure S7, S8 and S9, respectively—Supplementary Material). Furthermore, it is noteworthy that these metabolites have isomeric structures; therefore, for unambiguous confirmation, it is necessary to perform NMR spectroscopic characterization for each of the isolated metabolites.

**Figure 9:**
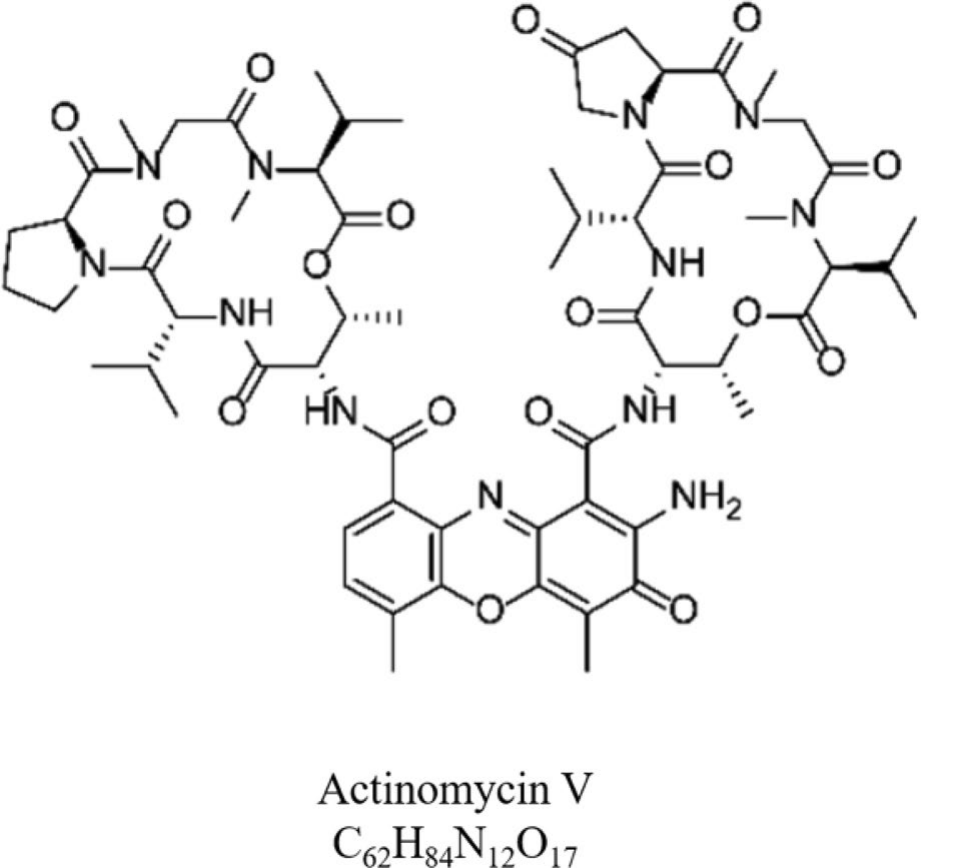
Chemical structure of actinomycin V.

The analysis of the *Streptomyces* sp. CMAA 1653 crude extract revealed a predominant peak at 25.30 min corresponding to mass spectra *m/z* 1,269 (Fig. 7b and Figure S10—Supplementary Material). The HRESIMS/MS spectrum showed the main product ions of *m/z* 956, *m/z* 857, *m/z* 657, *m/z* 558, *m/z* 459, *m/z* 399, and *m/z* 300 (Figure S11—Supplementary Material). These ions provided abundant structural information, and their identification was performed. HRESIMS/MS data were identical to those previously reported in the literature^44^. As a result of the fractionation procedures, FR-5, FR-6 and FR-7 were recovered, within the FR-7 portion as the main bioactive fraction (Fig. 5d and Table 1). Thus, the compound present in the fraction FR-7 was identified as actinomycin V (C_62_H_84_N_12_O_17_) (Fig. 9). The other fractions obtained by semi-preparative HPLC did not show bioactivity in the experiments carried out in this study.

## Discussion

The analysis of the rhizosphere microbiota in cold extreme habitats is crucial to understand the ecological roles and unlocking the biotechnological potential of these environments. Actinobacteria was also the dominant phylum in the rhizosphere of other *D. antarctica* retrieved from the maritime Antarctica region, as well as soils from cold and desert environments^45–48^. This prevalence may be explained by their spore-forming ability, UV radiation tolerance and other environmental adaptations that ensure survival in harsh conditions. Actinobacterial genera are known for their great potential to produce several bioactive substances^47,49,50^, although they also have the ability to degrade and use complex organic compounds and for their bioremediation of contaminated soils^51^. In fact, the predictive functional analysis using the Actinobacteria-derived 16S rRNA gene sequences showed enrichment of some functional categories related to carbohydrate metabolism, xenobiotic biodegradation and bioactive compound biosynthesis when compared with the total bacterial community. Vikram et al. (2016)^52^ also showed the genetic potential of Actinobacteria for C metabolism and membrane transport systems, which supports our PICRUSt functional predictions. The results obtained by 16S rRNA gene sequencing coupled to predictive functional analysis were useful to reveal diversified Actinobacteria pathways to produce biologically active metabolites. In doing so, we reported the presence of many pathways related to the biosynthesis of antibiotics such as streptomycin, novobiocin, phenylpropanoids, pyridine alkaloids, stilbenoids, neomycin, vancomycin, and tetracyclines, indicating a great bioactive potential of the bacterial community associated with *D. antarctica* (Table S5—Supplementary Material).

Despite advances in environmental sequencing (metagenomics) and single-cell genomics, the screening procedures of novel molecules remain largely related to the microorganisms culturing^53^. Thus, efforts to isolate new psychrotolerant strains were performed, resulting in a diverse collection of Actinobacteria revealed by the 16S rRNA gene sequencing. However, recent studies have found that discrepancies in terms of chemical and morphological features were possible in microorganisms belonging to same taxonomy, as determined for identical 16S rRNA gene sequences^54^. This finding was especially observed when studying bacteria from geographically distant origins or associated with different host species, in accordance to the ecovar concept^55–57^. Therefore, although the limitations of 16S rRNA gene have been widely recognized for screening of bioactive strains, we have assumed that the rhizosphere of *D. antarctica* could lead to high grade of specialism, with a lot of clonal populations. This fact was also observed in the 16S rRNA sequences of all isolated strains (data not shown), and supported by previous reports of Antarctic microbiota, which demonstrated that the type of habitat dramatically constrained the bacterial community composition^45,58^.

Although the Actinobacteria diversity has been underestimated through cultivation-dependent techniques, the isolation was able to access undiscovered species with the potential to produce novel bioactive compounds^59–61^. Some of them were not detected in microbiome analysis, possibly belonging to the rare community. Indeed, previous studies reported that cultivation techniques are still unable to access the real microbial diversity found in natural environments. The cultivation of recalcitrant microorganisms from extreme environments is difficult, especially the rhizosphere of Antarctic plants. However, the use of different culture media and growing conditions may be tested to improve the recovery of a major diversity of Actinobacteria members^14,62^. The low sequence identity registered in this study indicates the potential to identify novel species that require a polyphasic taxonomic approach for proper characterization and description. Based on phylogenetic, phenotypic and chemotaxonomic data, a new actinobacterial species isolated from *D. antarctica* was recently identified, named *Rhodococcus psychrotolerans* sp. nov^12^.

For current purposes, antiproliferative screening using *P. aphanidermatum* CMAA 243^T^ as a model organism resulted in 17 bioactive strains. The bioassay was followed the protocol successfully applied to the primary screening against the Oomycota *Pythium,* which contain in its hyphal walls and cell membrane, proteins, lipids and sterols such as cholesterol, compounds which resemble cancer cells. The advantage of this method is the simplicity, low cost and practicality^63^. Among these strains, *Streptomyces* sp. CMAA 1527 and *Streptomyces* sp. CMAA 1653 stood out, showing a high antiproliferative index for human tumour cells. According to the phylogenetic reconstruction and 16S rRNA gene similarity, *Streptomyces* sp. CMAA 1527 was closely related to *Streptomyces aurantiacus* NBRC 13017^T^ (99.37%), while *Streptomyces* sp. CMAA 1653 was related to *Streptomyces fildesensis* DSM 41987^T^ (99.72%). Interestingly, *Streptomyces fildesensis* DSM 41987^T^ was isolated from Antarctic soil and was recently presented as a prominent producer of antibiotic compounds^64^. Although both strains have been presented in the literature, they have not been listed as potential producers of antitumour compounds.

Cinerubin B is an important compound belonging to the anthracycline class. Anthracyclines are a special class of antibiotics widely used as antitumour agents, and the compounds daunomycin, doxorubicin, adriamycin, and aranciamycin anhydride have received great attention since their discovery^65^. The pronounced activity presented in the secondary metabolites of *Streptomyces* sp. CMAA 1653 can be clearly justified by the presence of actinomycin V (*m/z* 1,269.6170 u). The product ions at *m/z* 956, *m/z* 857, *m/z* 657, *m/z* 399 and *m/z* 300 corresponded to the loss of the Val-Pro-Sar-MeVal chain and were similar to those observed for actinomycin D^44^. Moreover, the product ion at *m/z* 558 indicated the presence of another Pro-Sar-MeVal residue in structure, as well as the product ion at *m/z* 459, which was characteristic of the core nucleus structure of actinomycins^44,66^. Actinomycins have been used as chemotherapeutic agents for the treatment of a variety of cancers and are produced by many *Streptomyce*s strains^24^. They are a family of bicyclic chromopeptide lactones that attach two pentapeptide lactones of nonribosomal origin. Its use dates approximately 70 years ago, acting under several types of malignant human tumours, including nephroblastoma (Wilms’ tumour) and childhood rhabdomyosarcoma^67–69^, and has attracted attention for its potential to assist in the development growth of multi-drug resistance strains and inhibition of HIV-1 reverse transcriptase^44,70,71^.

## Conclusions

This study reveals that the root-associated bacteria of *Deschampsia antarctica* Desv. from Antarctic ecosystems are a rich source of molecules with antitumour properties. The isolation methods employed in this study were able to retrieve Actinobacteria taxa that were not detected in microbiome analysis, showing that the combination of both strategies may be useful for recovering both rare and abundant members of the Actinobacteria communities for biotechnological exploration. The secondary metabolites of *Streptomyces* sp. CMAA 1527 and *Streptomyces* sp. CMAA 1653 showed valuable antiproliferative activities against human cancer cells and therefore can contribute significantly to the development of drugs of technological importance. Cinerubin B and actinomycin V were identified in the bioactive fractions of *Streptomyces* sp. CMAA 1527 and *Streptomyces* sp. CMAA 1653, respectively, but many other potential bioactive compounds can still be explored from Antarctic Actinobacteria. Based on these results, one of the two native Antarctic plants represents an attractive and unique model for the study of symbiosis and the discovery of antitumour lead compounds by the associated microbiome.

## Methods

### Sampling sites

Rhizospheric soil samples were collected (early summer Nov. 2014) at three different points (Morro da Cruz—62°05′04.3″ S/58°23′41.7″ W; Torre Meteoro Direito—62°05′10.4″ S/58°23′34.6″ W and Torre Meteoro Esquerdo—62°05′08.1″ S/58°23′36.6″ W) in Admiralty Bay, King George Island, South Shetland Islands, Antarctica. *Deschampsia antarctica* rhizospheric soil was obtained by removing plants from the soil (three plants for each collection point) and scraping 1–2 mm of soil adhering to the roots. Samples intended to actinobacteria isolation were kept at 4 °C and samples selected to genomic approach was stored at -20 °C until processed.

### Bacterial 16S rRNA metabarcoding sequencing

DNA extractions of the rhizosphere samples were performed using the PowerSoil DNA Isolation Kit (MoBio Laboratories—Carlsbad, CA, USA), according to the manufacturer's instructions. DNA yield and purity was evaluated by Qubit Fluorometric Quantification (Life Technologies—San Diego, CA, USA) and NanoDrop spectrophotometer (ThermoFischer Scientific—Waltham, MA, USA) (checking by the A260nm/280 nm and A260nm/230 nm ratios). The total DNA extracted from rhizosphere samples was ordered to the massive sequencing of the 16S rDNA gene for bacterial diversity analysis. The amplicons were obtained by the amplification of V6 16S rRNA hypervariable region^72^, using the primers 967F (5′-CAACGCGAAGAACCTTACC-3′) e 1193R (5′-CGTCRTCCCCRCCTTCC-3′)^73^, with an additional tag of five nucleotides added for each sample (https://vamps.mbl.edu/), according to Souza et al. (2017)^74^. The PCR products were pooled in equimolar ratio and purified by the SizeSelect EX E-Gel electrophoresis system (Life Technologies Corporation) for DNA size selection. The recovered fragments were further purified using the Agencourt AMPure XP kit (Beckman Coulter—Brea, CA, USA). Purified library product was quantified using the Qubit Fluorometric Quantification. The emulsion PCR procedure and sample enrichment were performed on the Ion OneTouch 2 system using the Ion Template PGM OT2 400 kit (Life Technologies Corporation). The V2 316 chip was used for sequencing as instructed in the Ion Torrent platform manual (Personal Genome Machine—PGM, Life Technologies Corporation).

### Statistical and bioinformatic analyses

Raw data obtained from Ion Torrent sequencing were converted to FASTA file and submitted to quality control (QC) using the Galaxy platform^75^, with the following QC parameters: quality score = 25; barcode size = 5; quality window = 50, and maximum number of homopolymers = 6. Sequences with low quality and lengths < 180 bp were removed. After QC, the remaining high-quality reads were analyzed by the Quantitative Insights Into Microbial Ecology (QIIME) version 1.9 software package^36^. Open reference operational taxonomic unit (OTU) were grouped at 97% sequence similarity OTU using UCLUST method and representative sequences of each OTU were taxonomically classified using the PyNAST alignment against GREENGENES (version gg_13_8) 16S reference database^37,76^. Chimeric sequences were detected and removed by the UCHIME algorithm^76^ before the construction of the OTU table. Afterward, all OTUs consisting of one single sequence (singletons), chloroplasts and non-bacterial sequences were removed before taxonomic classification.

The accurate predictive analysis was performed to infer the functional contribution of rhizosphere-associated Actinobacteria in comparison with the total community. Then, the PICRUSt tool^41^ was used to predict KEEG Orthology (KO) functional profiles of the bacterial community using the 16S rRNA dataset based on OTUs and reference genomes database^38–40^. For this, 16S reads affiliated to the Actinobacteria phylum were filtered after Greengenes database annotation. The final output of this workflow was quantified in terms of predicted function abundances per sample per OTU. Subsequently, the data were analyzed by the STAMP (Statistical Analysis of Metagenomic Profiles) software package v. 2.1.3^77^ to evaluate biologically meaningful differences between rhizosphere-associated Actinobacteria and the total bacterial community. Statistical inferences were performed by G-test (w/Yates) + Fisher's, DP method: Asymptotic-CC 0.95 and p-value > 0.05 filter.

### Isolation, culturing and identification of actinobacteria from rhizosphere

A pool of rhizosphere samples (0.25 g of each sample for three collected points; n = 9) was serially diluted (10^–4^, 10^–5^ and 10^–6^) in saline solution (NaCl 0.85%), and aliquots of 100 μL were plated on Starch Casein Agar (SCA)^78^, Humic Acid-Vitamin Agar (HVA)^79^ and Yeast Extract Malt Extract Agar (YEME)^80^. All media were supplemented with cycloheximide and nystatin (25 μg/mL) and incubated at 16 °C for 6 weeks. Isolates from the *Deschampsia antarctica* rhizosphere with the typical morphology of Actinobacteria colonies were taken from the selective isolation plates. The pure cultures obtained by repeated streaking were maintained on Glucose Yeast Extract Agar (GYEA) at 4 °C and as mixtures of mycelial fragments and spores in 20% glycerol (v/v) at − 80 °C.

Genomic DNA extraction, PCR amplification and sequencing methods were performed according to procedures adopted in Souza et al. (2017)^34^. The 16S rRNA consensus contigs were obtained using PhredPhrap/Consed^81^ and were aligned by ClustalW against nearest corresponding sequences using EzBioCloud^82^ and MEGA 7.0^42^. Phylogenetic trees were inferred using the neighbour-joining^83^ tree-making algorithm drawn from the MEGA 7.0 package. Topologies of the resultant trees were evaluated by bootstrap analysis^84^ based upon 1,000 replicates.

### Cultivation, extraction, and isolation of bioactive compounds

The metabolites of *Streptomyces* sp. CMAA 1527 and *Streptomyces* sp. CMAA 1653 were obtained by growing cultures in Potato Dextrose Broth on a rotating shaker (180 rpm, 16 °C) for 10 days. After growth, the mycelium was separated by centrifugation (7,000 rpm, 15 min, room temperature) followed by filtration (0.22 μm filter membrane). Each liquid culture medium was extracted with ethyl acetate (1:3 v/v), and the organic phase was evaporated to dryness under vacuum (Büchi Waterbath B-480). From each crude extract, at least three aliquots were separated, and two of them were subjected to antifungal (oomycete *Phytium aphanidermatum* CMAA 243^T^) and antiproliferative (human tumour and non-tumour cell lines) evaluations. Aliquots of each crude extract (650 mg) were fractionated by semipreparative HPLC on a Shimadzu LC system (Shimadzu, Kyoto, Japan) equipped with a CBM-20A controller, an LC-6AD pump, a DGU-20A5 degasser, and an SPD-20A UV–vis detector. The chromatographic separation was carried out using a C_18_ Zorbax Eclipse XDB column (250 × 9.4 mm, 5 μm; Agilent), and the mobile phase used was composed of 0.1% formic acid as system A and acetonitrile as system B. The gradient elution programme was 0—40 min from 30 to 100% of system B at a flow rate of 4 mL/min. The chromatograms were monitored using wavelengths at 320 and 500 nm.

### Screening of bioactive strains

#### Antifungal activity

Bioactive strains were screened as described by Santos and Melo (2016)^63^, using *Pythium. aphanidermatum* CMAA 243^T^ as a model organism. This bioassay was carried out as a primary screening for antitumour compounds from microorganisms. The antagonism assay was performed using sterile disc paper (0.5 cm diameter), crude extract solubilized in ethyl acetate (1 mg/mL) and potato dextrose agar medium. The plates were incubated at 28 °C for 24 h and evaluated according to the size of the inhibition zone, such as pronounced (+++), moderate (++), reduced (+) or absent (−).

#### Antiproliferative activity

The antiproliferative activity was investigated against a panel of human tumours [glioblastoma (U251), mamarian adenocarcinoma (MCF-7), ovarian multi-drug resistant adenocarcinoma (NCI/ADR-RES), large cell carcinoma of lung (NCI-H460), adenocarcinoma of kidney (786-0), ovarian adenocarcinoma (OVACAR-03), colon adenocarcinoma (HT-29) and chronic myeloid leukaemia (K-562)] and non-tumour (HaCat, immortalized keratinocytes) cell lines growing in complete medium [RPMI 1640 supplemented with 5% foetal bovine serum and 1% penicillin/streptomycin mixture (1,000 UI:1,000 μg/mL)] at 37 °C and 5% CO_2_ in a humidified atmosphere. Human tumour and the human non-tumoural cell lines were provided by the Frederick Cancer Research & Development Center (National Cancer Institute, Frederick, MD, USA), and Dr. Ricardo Della Colleta (University of Campinas).

The in vitro assay was performed as described by Monks et al. (1991)^85^. First, all extracts were screened against three tumour cell lines (U251, MCF-7 and NCI-H460). Then, the most promising extracts were evaluated against the complete panel. For both experiments, each extract was solubilized in dimethyl sulfoxide (100 mg/mL) followed by serial dilution in complete medium affording the final concentrations (0.25, 2.5, 25 and 250 μg/mL). Doxorubicin was used as a positive control at final concentrations of 0.025, 0.25, 2.5 and 25 μg/mL and was diluted following the same protocol.

Each cell line was grown in 96-well plates (100 μL cells/well) for 24 h and exposed for to the extracts, doxorubicin (positive control) or complete medium (negative control) for 48 h. Before (T_0_ plate) and after (T_1_ plates) sample addition, cells were fixed with 50% trichloroacetic acid dyed with sulforhodamine B 0.4% in acetic acid 0.1%, and colorimetric evaluation was recorded at 540 nm. The difference in absorbance between the untreated cells before (T_0_ plate) and after (T_1_ plates) sample addition represented 100% cell proliferation. Negative values represented a reduction in the cell population related to the T_0_ plate. The cell proliferation data were analysed using Origin 8.0 software (OriginLab Corporation) using sigmoidal regression to calculate the TGI and GI_50_ values (sample concentrations required total cell growth inhibition or to elicit 50% of cell death, respectively).

### LC–MS analysis

The compounds present in the bioactive fractions were analysed using a Waters ACQUITY UPLC *H-Class* system coupled to the Xevo TQ-S tandem quadrupole (Waters Corporation, Milford, MA, USA) mass spectrometer with a Z-spray source operating in the positive mode and to the diode arrangement detector (DAD) from 220 to 600 nm, according to Crevelin et al. (2014)^31^. The samples were solubilized in MeOH and injected (5 µL) into a Zorbax Eclipse XDB-C18 column (150 × 4.6 mm, 3.5 µm particle size—Agilent, Santa Clara, CA, USA); the mobile phase used for gradient elution consisted of 0.1% formic acid as system A and acetonitrile with 0.1% formic acid as system B. The flow rate was 0.5 mL/min, and the gradient elution programme started with 30% B, increased to 90% B in the following 25 min, remained at 90% B for 5 min, and returned to the initial condition within the following 5 min. The source and operating parameters were optimized as follows: capillary voltage, 3.2 kV; cone voltage, 40 V; source offset, 60 V; Z-spray source temperature, 150 °C; desolvation temperature (N_2_), 350 °C; desolvation gas flow, 800 L/h (mass range from *m/z* 150 − 1,200).

### High-resolution mass spectrometry analysis

The exact mass of the compounds present in the bioactive fractions was determined by high-resolution mass spectrometry (HRESIMS)^31,86^ on a mass spectrometer microTOFII‑ESI‑Q‑TOF (Bruker Daltonics, Billerica, MA, USA) employing an infusion pump (Kd Scientific, Holliston, MA, USA) at a flow rate of 100 µL/min. The voltage of the capillary was 3,500 V, and the voltage of the end plate was − 400 V in positive ionization mode. The source and operating parameters were optimized as follows: drying gas temperature (N_2_), 250 °C; a flow rate of 4 mL/min and a pressure of 0.4 bar. The tandem mass spectrometry experiments (MS/MS) with collision-induced dissociation were carried out using N_2_ as the collision gas on the selected precursor ion at collision energy values ranging from 10 to 50 eV. For internal calibration, a solution of sodium trifluoroacetic acid (Na-TFA) at a concentration of 1 mg/mL was used. Data acquisition and analysis were performed using Compass Data Analysis 4.1 software (Bruker Daltonics Corporation).

### NMR analysis

Nuclear magnetic resonance (NMR) measurements were carried out on a Bruker DRX 500 spectrometer (Bruker Daltonics Corporation) operating at 500 MHz for ^1^H and 125 MHz for ^13^C (δ in parts per million relative to Me4Si, *J* in Hertz). The isolated sample (5 mg) was dissolved in deuterated chloroform (CDCl_3_, Sigma-Aldrich) and analysed by 1D and 2D NMR using a Shigemi 5 mm NMR microtube, as described in Crevelin et al. (2013)^86^.

## Supplementary information

Supplementary information.
