# Supplementary material for: Actinobacteria from Antarctica as a source for anticancer discovery
Source: Sci Rep. 2020 Aug 17;10:13870. doi: 10.1038/s41598-020-69786-2 (PMC7431910; doi:10.1038/s41598-020-69786-2)
Supplement: Supplementary file 1 — Supplementary information. [file 41598_2020_69786_MOESM1_ESM.doc]

**Actinobacteria from Antarctica as a Source for Anticancer Discovery**

**Authors:** Leonardo Jose Silva^1^; Eduardo José Crevelin^2^; Danilo Tosta Souza^2^; Gileno Vieira Lacerda-Júnior^3^; Valeria Maia de Oliveira^4^; Ana Lucia Tasca Gois Ruiz^5^; Luiz Henrique Rosa^6^; Luiz Alberto Beraldo Moraes^2^; Itamar Soares Melo*^3^

**Supplementary Material:**

**Table S1:** Summary of the data processed by QIIME and PICRUSt tools

| **Dataset** | **Raw**  **reads** | **After**  **QC** | **Number of OTUs** | **Predicted gene abundance** |
| --- | --- | --- | --- | --- |
| TMD 1.1 | 23.337 | 22.729 | 20.910 | 3.334.501 |
| TMD 1.2 | 23.333 | 22.647 | 22.194 | 4.340.979 |
| TMD 1.3 | 43.772 | 40.684 | 36.615 | 5.789.574 |
| TME 2.1 | 38.945 | 36.529 | 32.510 | 7.093.661 |
| TME 2.2 | 30.368 | 28.770 | 25.893 | 4.769.886 |
| TME 2.3 | 31.530 | 28.269 | 26.572 | 4.264.344 |
| MDC 3.1 | 25.468 | 22.220 | 21.109 | 2.993.413 |
| MDC 3.2 | 27.792 | 25.332 | 23.812 | 3.458.017 |
| MDC 3.3 | 22.889 | 21.996 | 20.016 | 3.130.941 |

- OTUs assigned at 97% similarity. KEEG orthology (KO) function predicted by PICRUSt analysis (Langille et al., 2013).

**
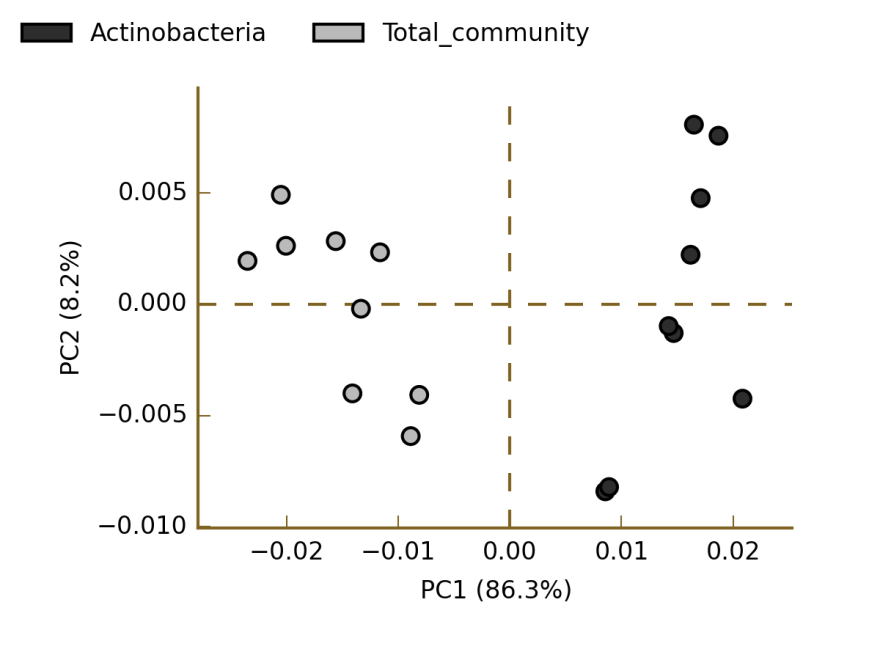
**

**Figure S1:** Principal component analysis (PCA) comparing PICRUSt-inference of KEEG orthology (KO) functional traits derived from the total *Deschampsia antarctica* bacterial community (grey circles) and Actinobacteria (black circles) (Kanehisa et al., 2019; Kanehisa, 2019; Kanehisa; Goto, 2000). Traits were normalized as percentages of total genes assigned for each dataset.

**Table S2:** Taxonomic relationship between isolated strains of Deschampsia antarctica Desv. rhizosphere and nearst type strains.

| **Strains** | **Family** | **Nearest type strain** | **Identity (%)** |  | **GenBank** |
| --- | --- | --- | --- | --- | --- |
| CMAA 1515 | Streptomycetaceae | *Streptacidiphilus albus* | 98.99 |  | MH241008 |
| CMAA 1516 | Micromonosporacea | *Pilimelia columellifera subsp. pallida* | 98.37 |  | MH241011 |
| CMAA 1517 | Streptomycetaceae | *Streptomyces xanthophaeus* | 99.61 |  | MH241013 |
| CMAA 1518 | Streptomycetaceae | *Streptomyces avidinii* | 99.69 |  | MH241014 |
| CMAA 1519 | Streptomycetaceae | *Streptomyces xanthophaeus* | 99.65 |  | MH241015 |
| CMAA 1520 | Streptomycetaceae | *Streptomyces luridiscabiei* | 99.93 |  | MH241016 |
| CMAA 1521 | Streptomycetaceae | *Streptomyces globisporus* | 100 |  | MH241017 |
| CMAA 1522 | Streptomycetaceae | *Streptomyces coelicoflavus* | 100 |  | MH241019 |
| CMAA 1523 | Streptomycetaceae | *Streptomyces griseoaurantiacus* | 99.92 |  | MH241020 |
| CMAA 1524 | Streptomycetaceae | *Streptomyces olivaceus* | 99.93 |  | MH241021 |
| CMAA 1525 | Streptomycetaceae | *Streptomyces diastaticus subsp. ardesiacus* | 99.65 |  | MH241022 |
| CMAA 1526 | Streptomycetaceae | *Streptomyces parvulus* | 99.86 |  | MH241023 |
| CMAA 1527 | Streptomycetaceae | *Streptomyces glomeroaurantiacus* | 99.37 |  | MH241026 |
| CMAA 1528 | Streptomycetaceae | *Streptomyces alni* | 98.25 |  | MH241029 |
| CMAA 1529 | Nocardiaceae | *Rhodococcus yunnanensis* | 99.22 |  | MH241030 |
| CMAA 1530 | Nocardiaceae | *Rhodococcus trifolii* | 98.79 |  | MH241033 |
| CMAA 1531 | Tsukamurellaceae | *Tsukamurella pulmonis* | 99.93 |  | MH241034 |
| CMAA 1532 | Nocardiaceae | *Nocardia asteroides* | 100 |  | MH241035 |
| CMAA 1533 | Nocardiaceae | *Rhodococcus baikonurensis* | 96.91 |  | KY317932 |
| CMAA 1536 | Streptomycetaceae | *Streptacidiphilus carbonis* | 98.96 |  | MH241010 |
| CMAA 1537 | Micrococcaceae | *Arthrobacter cryoconiti* | 98.53 |  | MH241018 |
| CMAA 1538 | Streptomycetaceae | *Streptomyces violaceochromogenes* | 99.50 |  | MH241025 |
| CMAA 1539 | Nocardiaceae | *Nocardia ninae* | 98.18 |  | MH241036 |
| CMAA 1650 | Mycobacteriaceae | *Mycobacterium hodleri* | 98.85 |  | MH241031 |
| CMAA 1651 | Micrococcaceae | *Pseudarthrobacter siccitolerans* | 99.22 |  | MH241028 |
| CMAA 1652 | Nocardioidaceae | *Kribbella ginsengisoli* | 99.28 |  | MH241024 |
| CMAA 1653 | Streptomycetaceae | *Streptomyces fildesensis* | 99.72 |  | MH241012 |
| CMAA 1654 | Streptomycetaceae | *Streptomyces fildesensis* | 99.43 |  | MH241009 |
| CMAA 1703 | Streptomycetaceae | *Streptomyces ossamyceticus* | 98.90 |  | MH241027 |
| CMAA 1704 | Micromonosporaceae | *Actinoplanes brasiliensis* | 98.69 |  | MH241032 |


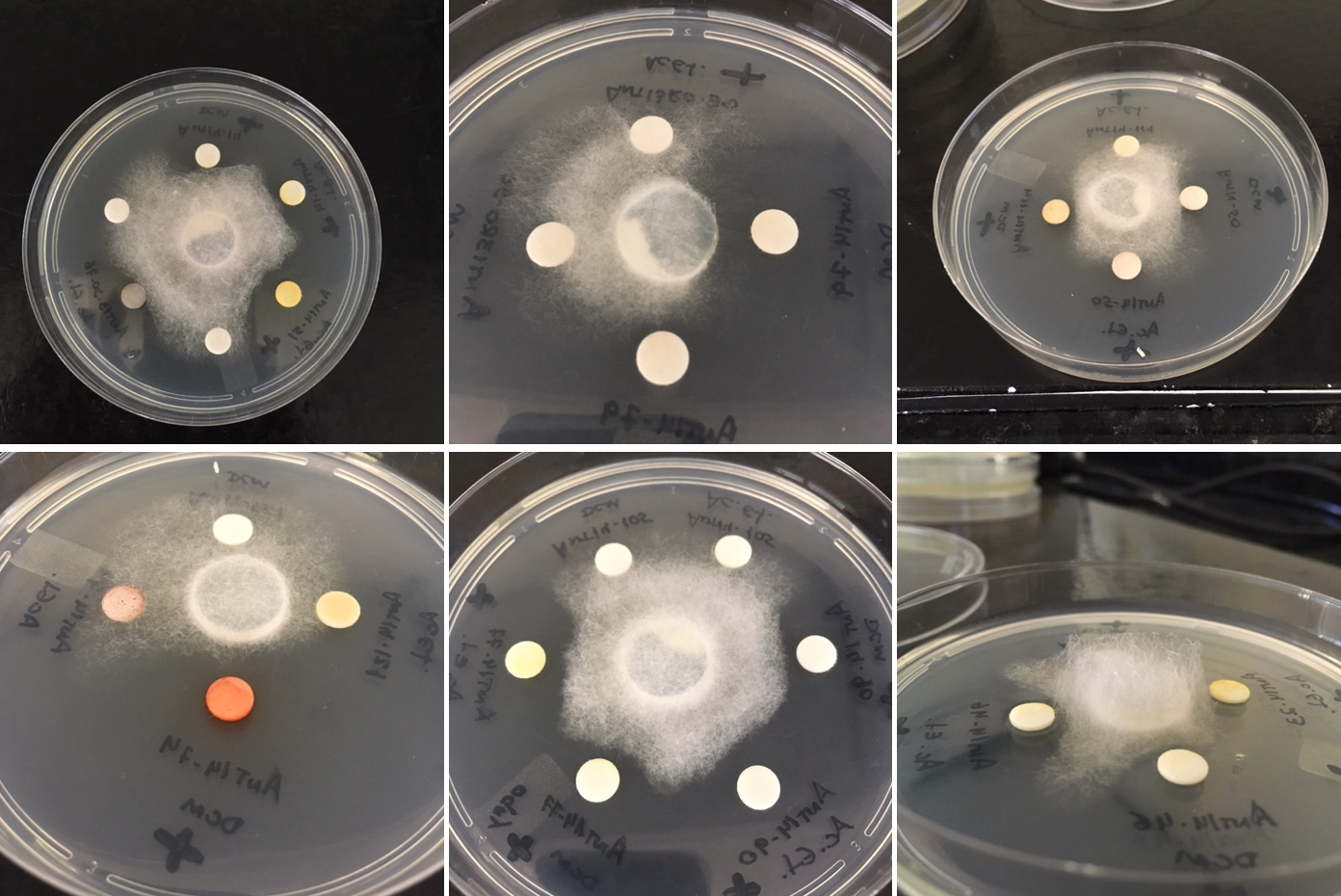


**Figure S2:** Antagonism test. *Phytium aphanidermatum* CMAA 243^T^ against actinobacterial crude extracts

**Table S3:** Qualitative inference - Antagonism potential of crude extracts against *Phytium aphanidermatum* CMAA 243^T^

| **Strains:** | **Activity** |
| --- | --- |
| CMAA 1515 | + + |
| CMAA 1516 | + |
| CMAA 1517 | + + |
| CMAA 1518 | + |
| CMAA 1519 | + + |
| CMAA 1520 | + |
| CMAA 1521 | - |
| CMAA 1522 | + + |
| CMAA 1523 | + + |
| CMAA 1524 | + + |
| CMAA 1525 | + |
| CMAA 1526 | - |
| CMAA 1527 | + + + |
| CMAA 1528 | - |
| CMAA 1529 | - |
| CMAA 1530 | + |
| CMAA 1531 | - |
| CMAA 1532 | + + |
| CMAA 1533 | - |
| CMAA 1536 | + + |
| CMAA 1537 | - |
| CMAA 1538 | - |
| CMAA 1539 | - |
| CMAA 1650 | - |
| CMAA 1651 | - |
| CMAA 1652 | - |
| CMAA 1653 | + + + |
| CMAA 1654 | + |
| CMAA 1703 | + + |
| CMAA 1704 | - |

Legend: (+ + +) pronounced; (+ +) moderate; (+) reduced and (-) absent activity

**Figure S3:** Mass spectrum of cinerubin B obtained by HRESIMS.


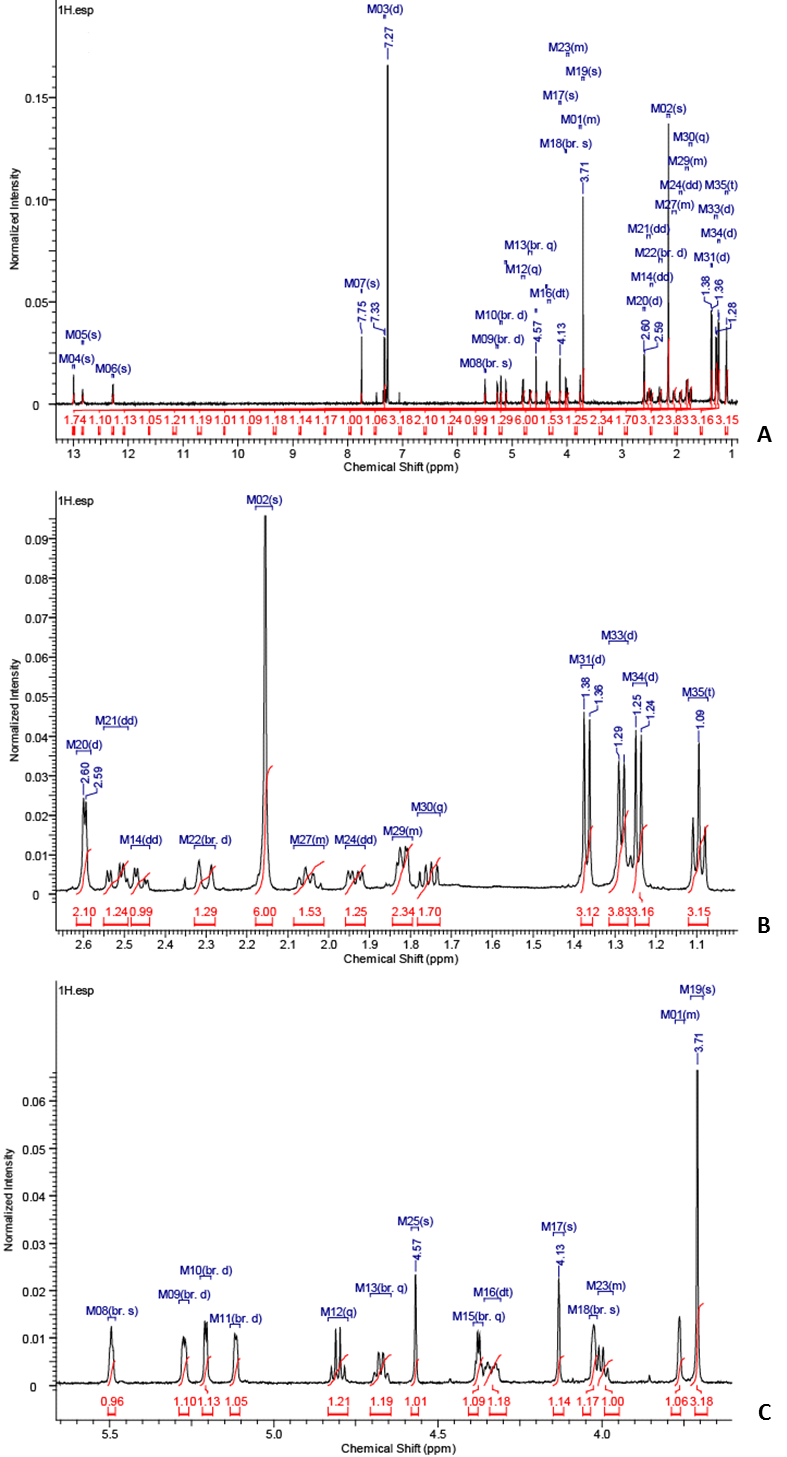


**Figure S4:** NMR spectra of cinerubin B.


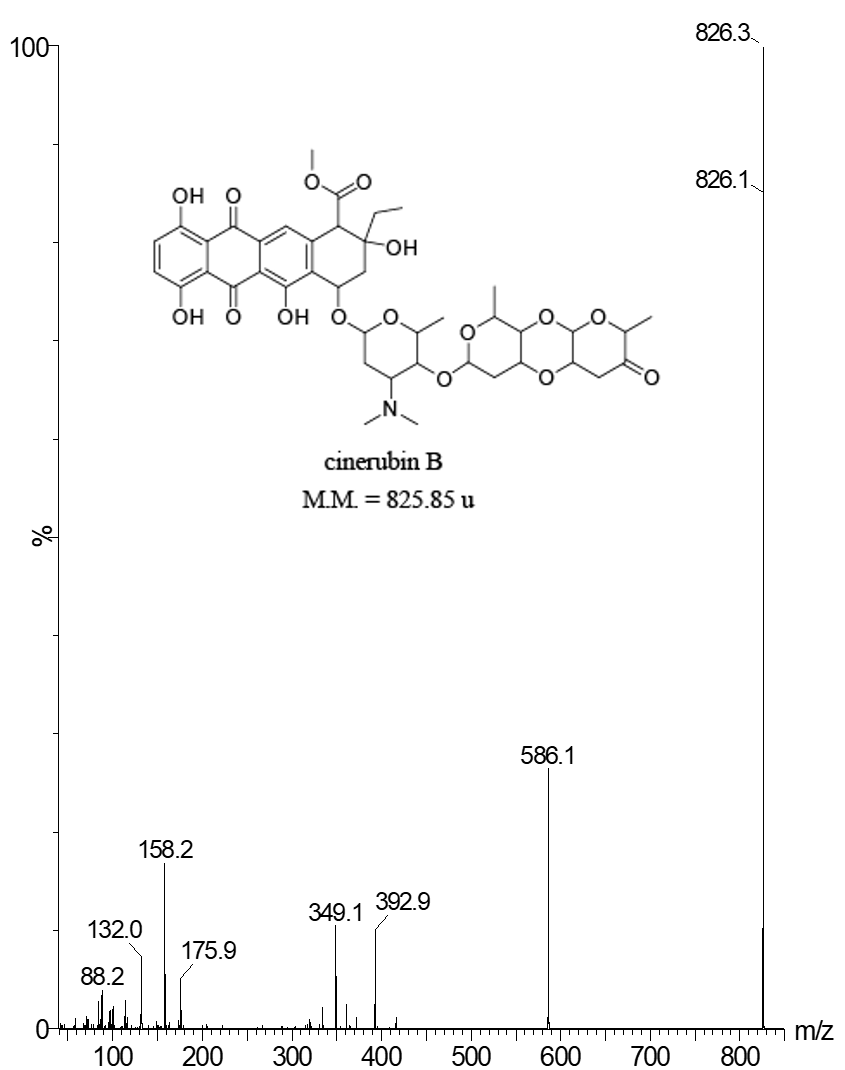


**Figure S5:** LC-MS/MS spectrum from the íon of *m/z* 826 (produced by *Streptomyces* sp. CMAA 1527)

**Table S4:** ^1^H NMR, ^13^C NMR chemical shifts and the 2D correlations observed for cinerubin B.

| **Position** | ***δ*C (ppm)*** | ***δ*H (ppm)*** | **HMBC*** | **COSY*** |
| --- | --- | --- | --- | --- |
| 1 | 157.9 | --- | --- | --- |
| 1-OH | --- | 12.99 (s, 1H) | C-1, C12a | --- |
| 2 | 129.8 | 7.33 (d, *J*=4.4 Hz, 2H) | C-1, C-12a | H-3 |
| 3 | 129.8 | 7.33 (d, *J*=4.4 Hz, 2H) | C-4, C-4a | H-2 |
| 4 | 157.9 | --- | --- | --- |
| 4-OH | --- | 12.28 (s, 1H) | C-4, C-4a, C-5 | --- |
| 4a | 112.4 | --- | --- | --- |
| 5 | 190.6 | --- | --- | --- |
| 5a | 114.8 | --- | --- | --- |
| 6 | 162.2 | --- | --- | --- |
| 6-OH | --- | 12.83 (s, 1H) | C-5a, C-6 | --- |
| 6a | 131.3 | --- | --- | --- |
| 7 | 70.4 | 5.27 (dl, *J*=2.5 Hz, 1H) | C-6, C-6a, C-1’, C-9, C-10a | H-8 |
| 8a | 33.7 | 2.52 (dd, *J*=15.3 Hz, 4.0 Hz, 1H) | C-6a, C-9, C-10 | H-7, H-8b |
| 8b | 33.7 | 2.30 (dl, *J*=15.3 Hz, 1H) | C-6a, C-9, C-10 | H-8a |
| 9 | 71.7 | --- | --- | --- |
| 10 | 56.9 | 4.13 (s, 1H) | C-6a, C-8, C-9, C-10a, C-11, C-13 | --- |
| 10a | 142.3 | --- | --- | --- |
| 11 | 120.3 | 7.75 (s, 1H) | C-5, C-5a, C-6a, C-10, C-12 | --- |
| 11a | 130.4 | --- | --- | --- |
| 12 | 185.9 | --- | --- | --- |
| 12a | 112.4 | --- | --- | --- |
| 13 | 171.2 | --- | --- | --- |
| 14 | 52.6 | 3.71 (s, 3H) | C-13 | --- |
| 15 | 32.0 | 1.76 (q, *J*=7.0 Hz, 2H) | C-8, C-9, C-16 | H-16 |
| 16 | 6.6 | 1.09 (t, *J*=7.0 Hz, 3H) | C-9, C-15 | H-15 |
| 1’ | 101.3 | 5.49 (sl, 1H) | C-5’ | H-2’ |
| 2’ | 29.2 | 1.82 (m, 2H) | C-1’, C-3’, C-4’ | H-3’ |
| 3’ | 61.4 | 2.05 (m, 1H) | N-(CH_3_)_2_ | H-2’ |
| 4’ | 74.1 | 3.76 (m, 1H) | C-2’, C-3’, C-1’’ | H-3’ |
| 5’ | 68.0 | 4.00 (m, 1H) | C-4’, C-6’ | H-6’ |
| 6’ | 17.7 | 1.28 (d, *J*=6.5 Hz, 3H) | C-2’, C-4’, C-5’ | H-5’ |
| N-(CH_3_)_2_ | 43.1 | 2.16 (s, 6H) | C-3’, N-CH_3_ | --- |
| 1’’ | 98.9 | 5.12 (dl, *J*=2.9 Hz, 1H) | C-4’, C-4’’, C-5’’ | H-2’’ |
| 2a’’ | 26.8 | 2.46 (dd, *J*=12.2 Hz, 3.6 Hz, 1H) | C-4’’ | H-2b’’, H-1’’, H-3’’ |
| 2b’’ | 26.8 | 1.94 (dd, *J*=12.2 Hz, 4.0 Hz, 1H) | C-1’’, C-4’’ | H-2a’’, H-3’’ |
| 3’’ | 67.1 | 4.33 (dt, *J*=12.2 Hz, 3.6 Hz, 1H) | --- | H-2’’ |
| 4’’ | 66,7 | 4.03 (sl, 1H) | C-6’’ | H-3’’ |
| 5’’ | 65.3 | 4.67 (ql, *J*=6.5 Hz, 1H) | C-3’’, C-6’’ | H-6’’ |
| 6’’ | 16.0 | 1.24 (d, *J*=6.5 Hz, 3H) | C-4’’, C5’’ | H-5’’ |
| 1’’’ | 91.4 | 5.21 (dl, *J*=2.9 Hz, 1H) | C-4’’, C-2’’’, C5’’’ | H-2’’’ |
| 2’’’ | 62.7 | 4.38 (ql, *J*=2.9 Hz, 1H) | C-4’’’ | H-1’’’ |
| 3’’’ | 39.5 | 2.60 (d, *J*=2.9 Hz, 2H) | C-1’’’, C-2’’’, C-4’’’ | H-2’’’ |
| 4’’’ | 208.2 | --- | --- | --- |
| 5’’’ | 77.8 | 4.80 (q, *J*=6.7 Hz, 1H) | C-4’’’, C-6’’’ | H-6’’’ |
| 6’’’ | 16.0 | 1.37 (d, *J*=6.7 Hz, 3H) | C-4’’’, C-5’’’ | H-5’’’ |

**Legend:** *Chemical shifts of hydrogen atoms were obtained from the ^1^H NMR spectrum. The two-dimensional ^1^H-^1^H-COSY, ^1^H-^13^C-HSQC and ^1^H-^13^C-HMBC spectra were also used in these correlations.


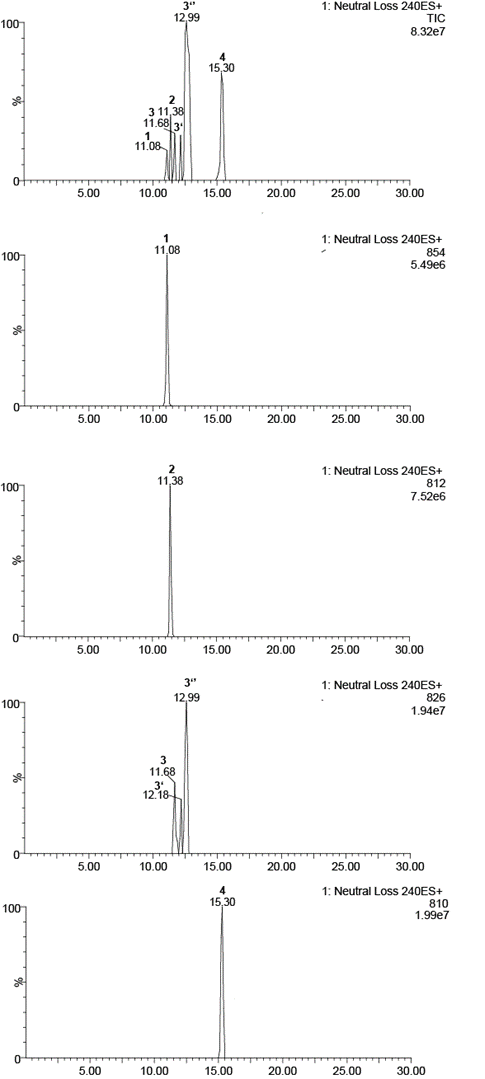


**Figure S6:** Chromatograms (XIC) obtained from the neutral loss experiment


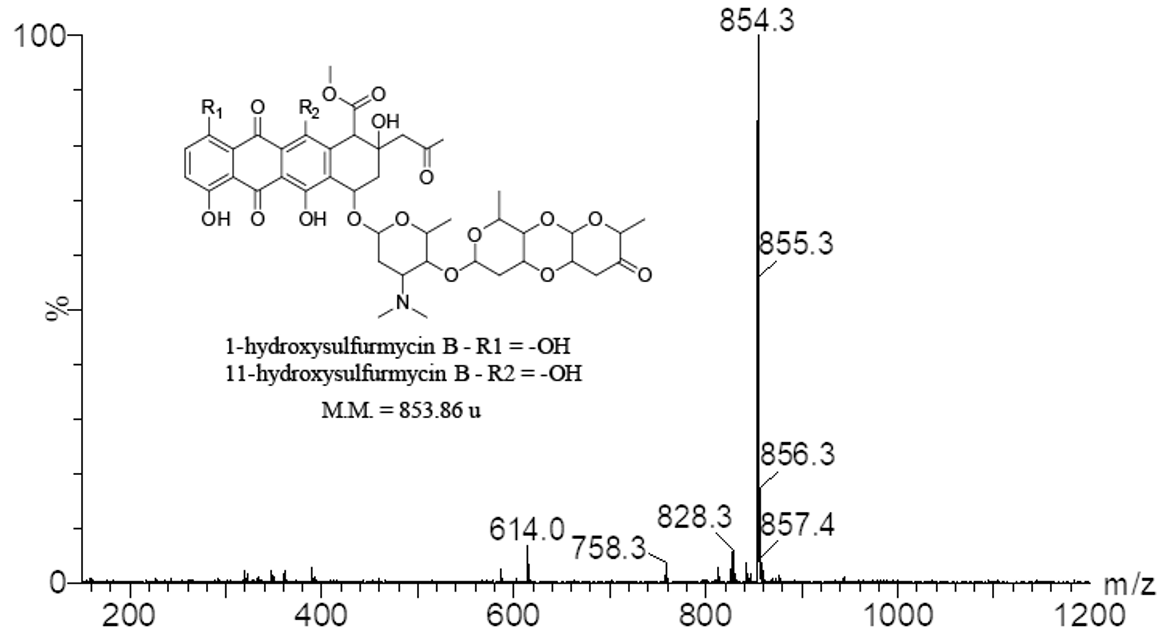


**Figure S7:** LC-MS chromatogram – *m/z* 854 (produced by *Streptomyces* sp. CMAA 1527)


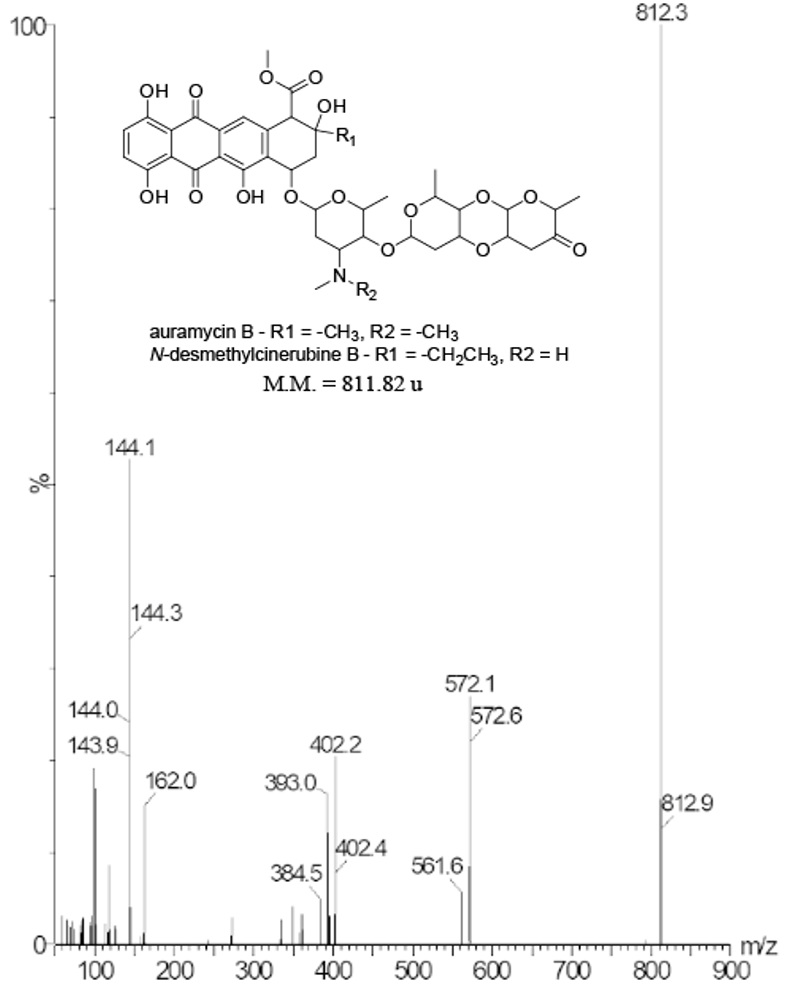


**Figure S8:** LC-MS/MS chromatogram – m/z 812 (produced by *Streptomyces* sp. CMAA 1527)


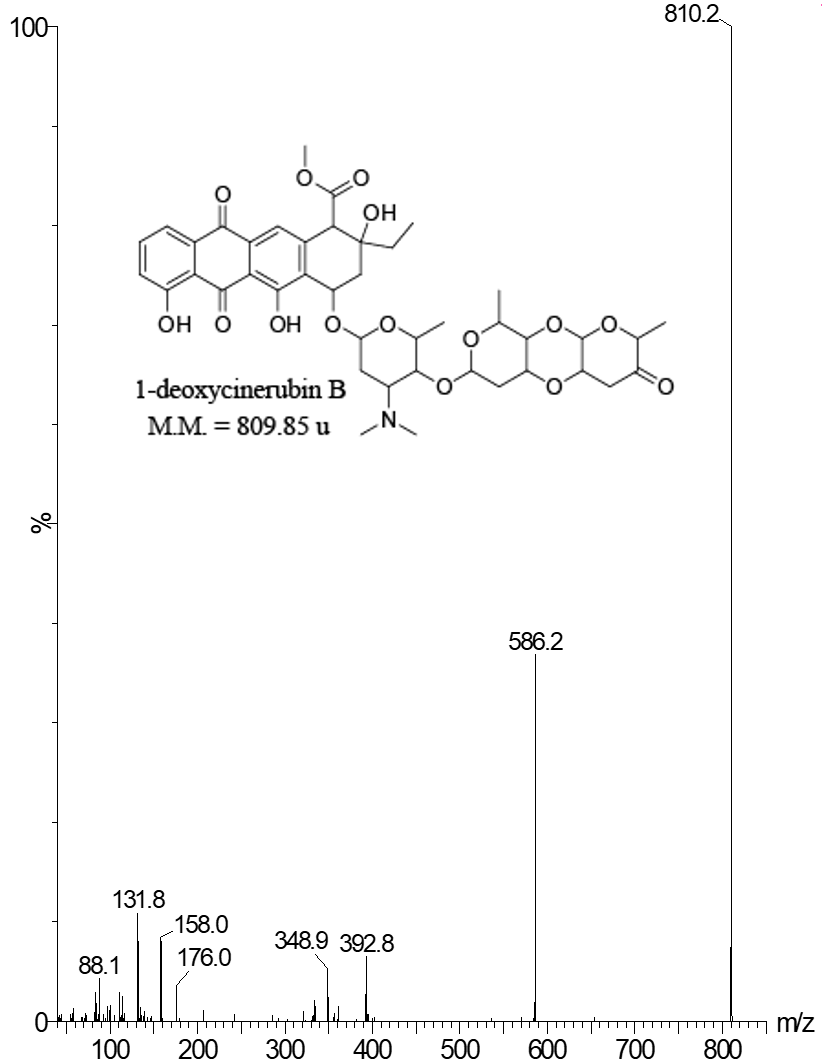


**Figure S9:** LC-MS/MS spectrum from the íon of *m/z* 810 (produced by *Streptomyces* sp. CMAA 1527)

**Figure S10:** Mass spectrum of actinomycin V obtained by HRESIMS.

**Figure S11:** Product ions spectrum of actinomycin V obtained by HRESIMS/MS.

**Table S5: Prediction functional content from 16S rRNA amplicon abundance data**

|  | **MDC_1** | **MDC_2** | **MDC_3** | **TMD_1** | **TMD_2** | **TMD_3** | **TME_1** | **TME_2** | **TME_3** |  |  |
| --- | --- | --- | --- | --- | --- | --- | --- | --- | --- | --- | --- |
| Amino sugar and nucleotide sugar metabolism | 7,43% | 7,61% | 7,71% | 8,01% | 7,84% | 7,54% | 7,75% | 7,87% | 7,88% |  |  |
| Ascorbate and aldarate metabolism | 1,17% | 1,17% | 1,10% | 1,14% | 1,20% | 1,16% | 1,09% | 1,08% | 1,06% |  |  |
| beta-Lactam resistance | 0,21% | 0,20% | 0,19% | 0,23% | 0,27% | 0,25% | 0,16% | 0,15% | 0,18% |  |  |
| Betalain biosynthesis | 0,02% | 0,02% | 0,02% | 0,03% | 0,04% | 0,02% | 0,02% | 0,02% | 0,04% |  |  |
| Biosynthesis of 12-, 14- and 16-membered macrolides | 0,03% | 0,02% | 0,02% | 0,02% | 0,01% | 0,03% | 0,02% | 0,03% | 0,02% |  |  |
| Biosynthesis of ansamycins | 0,47% | 0,48% | 0,43% | 0,49% | 0,47% | 0,45% | 0,42% | 0,40% | 0,43% |  |  |
| Biosynthesis of siderophore group nonribosomal peptides | 0,33% | 0,34% | 0,33% | 0,34% | 0,41% | 0,39% | 0,35% | 0,35% | 0,33% |  |  |
| Biosynthesis of type II polyketide backbone | 0,02% | 0,02% | 0,02% | 0,02% | 0,00% | 0,02% | 0,00% | 0,00% | 0,03% |  |  |
| Biosynthesis of type II polyketide products | 0,02% | 0,02% | 0,02% | 0,04% | 0,02% | 0,01% | 0,02% | 0,03% | 0,06% |  |  |
| Biosynthesis of vancomycin group antibiotics | 0,40% | 0,39% | 0,43% | 0,42% | 0,39% | 0,39% | 0,38% | 0,39% | 0,40% |  |  |
| Butanoate metabolism | 8,08% | 7,85% | 7,81% | 7,36% | 7,42% | 7,86% | 7,62% | 7,61% | 7,66% |  |  |
| Butirosin and neomycin biosynthesis | 0,42% | 0,44% | 0,48% | 0,43% | 0,50% | 0,52% | 0,46% | 0,49% | 0,50% |  |  |
| C5-Branched dibasic acid metabolism | 2,29% | 2,34% | 2,38% | 2,39% | 2,30% | 2,34% | 2,37% | 2,32% | 2,37% |  |  |
| Caffeine metabolism | 0,03% | 0,03% | 0,03% | 0,02% | 0,03% | 0,03% | 0,03% | 0,04% | 0,02% |  |  |
| Carotenoid biosynthesis | 0,36% | 0,35% | 0,35% | 0,41% | 0,32% | 0,34% | 0,30% | 0,28% | 0,39% |  |  |
| Citrate cycle (TCA cycle) | 6,21% | 6,22% | 6,40% | 6,24% | 6,06% | 6,16% | 6,42% | 6,50% | 6,42% |  |  |
| Clavulanic acid biosynthesis | 0,00% | 0,00% | 0,00% | 0,00% | 0,00% | 0,00% | 0,00% | 0,00% | 0,00% |  |  |
| Flavone and flavonol biosynthesis | 0,07% | 0,07% | 0,05% | 0,09% | 0,08% | 0,08% | 0,06% | 0,05% | 0,04% |  |  |
| Flavonoid biosynthesis | 0,20% | 0,19% | 0,21% | 0,20% | 0,21% | 0,22% | 0,21% | 0,23% | 0,22% |  |  |
| Fructose and mannose metabolism | 4,06% | 4,20% | 4,12% | 4,17% | 4,30% | 3,98% | 4,23% | 4,25% | 4,04% |  |  |
| Galactose metabolism | 3,10% | 3,26% | 3,27% | 3,46% | 3,73% | 3,49% | 3,51% | 3,60% | 3,56% |  |  |
| Geraniol degradation | 2,75% | 2,54% | 2,63% | 2,27% | 2,31% | 2,76% | 2,42% | 2,49% | 2,53% |  |  |
| Glycolysis / Gluconeogenesis | 7,99% | 8,12% | 8,26% | 7,89% | 8,22% | 8,10% | 8,37% | 8,52% | 8,34% |  |  |
| Glyoxylate and dicarboxylate metabolism | 6,72% | 6,61% | 6,10% | 6,32% | 6,51% | 6,04% | 6,03% | 5,53% | 5,77% |  |  |
| Indole alkaloid biosynthesis | 0,02% | 0,02% | 0,03% | 0,03% | 0,04% | 0,02% | 0,02% | 0,02% | 0,04% |  |  |
| Inositol phosphate metabolism | 1,50% | 1,52% | 1,55% | 1,51% | 1,74% | 1,74% | 1,55% | 1,58% | 1,74% |  |  |
| Isoflavonoid biosynthesis | 0,01% | 0,02% | 0,02% | 0,01% | 0,03% | 0,03% | 0,02% | 0,02% | 0,02% |  |  |
| Isoquinoline alkaloid biosynthesis | 0,34% | 0,35% | 0,32% | 0,39% | 0,43% | 0,31% | 0,28% | 0,25% | 0,30% |  |  |
| Limonene and pinene degradation | 2,80% | 2,62% | 2,74% | 2,41% | 2,47% | 3,13% | 2,63% | 2,76% | 2,69% |  |  |
| Novobiocin biosynthesis | 0,93% | 0,94% | 0,91% | 0,95% | 0,97% | 0,89% | 0,89% | 0,88% | 0,91% |  |  |
| Penicillin and cephalosporin biosynthesis | 0,50% | 0,50% | 0,51% | 0,58% | 0,56% | 0,50% | 0,51% | 0,47% | 0,52% |  |  |
| Pentose and glucuronate interconversions | 3,11% | 3,22% | 3,06% | 3,55% | 3,55% | 3,23% | 3,23% | 3,21% | 3,21% |  |  |
| Pentose phosphate pathway | 4,96% | 5,07% | 4,98% | 5,22% | 5,27% | 5,00% | 5,04% | 4,95% | 5,02% |  |  |
| Phenylpropanoid biosynthesis | 0,85% | 0,87% | 0,83% | 0,92% | 1,01% | 0,89% | 0,98% | 0,97% | 0,84% |  |  |
| Polyketide sugar unit biosynthesis | 1,10% | 1,06% | 1,16% | 1,12% | 0,97% | 1,00% | 1,07% | 1,08% | 1,09% |  |  |
| Prenyltransferases | 2,45% | 2,47% | 2,59% | 2,51% | 2,36% | 2,43% | 2,55% | 2,61% | 2,63% |  |  |
| Propanoate metabolism | 7,58% | 7,31% | 7,27% | 7,01% | 6,81% | 7,32% | 6,98% | 6,97% | 7,09% |  |  |
| Pyruvate metabolism | 8,69% | 8,61% | 8,49% | 8,63% | 8,07% | 8,15% | 8,52% | 8,34% | 8,27% |  |  |
| Sesquiterpenoid biosynthesis | 0,00% | 0,00% | 0,00% | 0,00% | 0,00% | 0,00% | 0,00% | 0,00% | 0,00% |  |  |
| Starch and sucrose metabolism | 4,39% | 4,55% | 4,64% | 4,80% | 4,90% | 4,63% | 5,00% | 5,06% | 4,73% |  |  |
| Stilbenoid, diarylheptanoid and gingerol biosynthesis | 0,33% | 0,32% | 0,34% | 0,29% | 0,37% | 0,54% | 0,36% | 0,41% | 0,39% |  |  |
| Streptomycin biosynthesis | 2,27% | 2,28% | 2,46% | 2,34% | 2,31% | 2,39% | 2,39% | 2,46% | 2,55% |  |  |
| Terpenoid backbone biosynthesis | 3,78% | 3,80% | 3,88% | 3,71% | 3,59% | 3,74% | 3,91% | 4,00% | 3,86% |  |  |
| Tetracycline biosynthesis | 0,95% | 0,94% | 0,85% | 0,99% | 0,85% | 0,91% | 0,79% | 0,72% | 0,78% |  |  |
| Tropane, piperidine and pyridine alkaloid biosynthesis | 0,81% | 0,81% | 0,79% | 0,83% | 0,85% | 0,77% | 0,78% | 0,78% | 0,79% |  |  |
| Zeatin biosynthesis | 0,23% | 0,23% | 0,23% | 0,23% | 0,22% | 0,22% | 0,23% | 0,23% | 0,23% |  |  |

KEEG Orthology database at Kanehisa Laboratories (Kanehisa et al., 2019; Kanehisa, 2019; Kanehisa; Goto, 2000).
